# Scavenger receptor-C acts as a receptor for *Bacillus thuringiensis* vegetative insecticidal protein Vip3Aa and mediates the internalization of Vip3Aa via endocytosis

**DOI:** 10.1371/journal.ppat.1007347

**Published:** 2018-10-04

**Authors:** Kun Jiang, Xiao-yue Hou, Tong-tong Tan, Zhang-lei Cao, Si-qi Mei, Bing Yan, Jin Chang, Lu Han, Dan Zhao, Jun Cai

**Affiliations:** 1 Department of Microbiology, College of Life Sciences, Nankai University, Tianjin, China; 2 State Key Laboratory of Microbial Technology, Shandong University, Qingdao, China; 3 Britton Chance Center for Biomedical Photonics, Wuhan National Laboratory for Optoelectronics-Huazhong University of Science and Technology, Wuhan, Hubei, China; 4 College of Plant Protection, Hebei Agricultural University, Baoding, China; 5 Key Laboratory of Molecular Microbiology and Technology, Ministry of Education, Tianjin, China; 6 Tianjin Key Laboratory of Microbial Functional Genomics, Tianjin, China; Stanford University, UNITED STATES

## Abstract

The vegetative insecticidal proteins (Vip), secreted by many *Bacillus thuringiensis* strains during their vegetative growth stage, are genetically distinct from known insecticidal crystal proteins (ICPs) and represent the second-generation insecticidal toxins. Compared with ICPs, the insecticidal mechanisms of Vip toxins are poorly understood. In particular, there has been no report of a definite receptor of Vip toxins to date. In the present study, we identified the scavenger receptor class C like protein (Sf-SR-C) from the *Spodoptera frugiperda* (Sf9) cells membrane proteins that bind to the biotin labeled Vip3Aa, via the affinity magnetic bead method coupled with HPLC-MS/MS. We then certified Vip3Aa protoxin could interact with Sf-SR-C *in vitro* and *ex vivo*. In addition, downregulation of SR-C expression in Sf9 cells and *Spodoptera exigua* larvae midgut reduced the toxicity of Vip3Aa to them. Coincidently, heterologous expression of Sf-SR-C in transgenic *Drosophila* midgut significantly enhanced the virulence of Vip3Aa to the *Drosophila* larvae. Moreover, the complement control protein domain and MAM domain of Sf-SR-C are involved in the interaction with Vip3Aa protoxin. Furthermore, endocytosis of Vip3Aa mediated by Sf-SR-C correlates with its insecticidal activity. Our results confirmed for the first time that Sf-SR-C acts as a receptor for Vip3Aa protoxin and provides an insight into the mode of action of Vip3Aa that will significantly facilitate the study of its insecticidal mechanism and application.

## Author summary

*Bacillus thuringiensis* Vip3A has potential in control of Lepidopteran pest and has been used in transgenic plants. However, studies of the insecticidal mechanisms of Vip3A are rare, and none of their definite receptors have been reported so far, which seriously restricts the study of its insecticidal mechanism and application. This work identified and confirmed the scavenger receptor class C like protein (Sf-SR-C) acts as the receptor of Vip3Aa protoxin, demonstrated that Sf-SR-C mediates the toxicity of Vip3Aa to Sf9 cells in an internalized manner. These results extend our understanding of SR-C proteins in insects and explain the specificity of Vip3Aa insecticidal activity, which strongly support it as a safe biopesticide. More importantly, it suggests the insecticidal mechanism of Vip3Aa different from the well-known “pore formation” model, “signal transduction” model, as well as newly found “necrosis” model of Cry toxins, which will significantly promote the relevant study of Vip3Aa. Last but not least, because scavenger receptors play a crucial role in innate immunity, our results provide relevant insights into host-pathogen interactions.

## Introduction

Microbial insecticides, as substitutes for chemical pesticides, are alternatives for insect control in crops. *Bacillus thuringiensis* (Bt) is the most extensively used biopesticide worldwide due to its ability to produce insecticidal crystal proteins (Cry and Cyt toxins)[1–3]. The classical pore-forming model is the widely accepted mode of action of the three-domain crystal protein (3d-Cry) [1]. A signaling pathway model of the Cry toxin’s action has also been reported [4, 5]. Recently, Fengjuan et al. showed Cry6Aa could trigger the *Caenorhabditis elegans* death by necrosis signaling pathway [6]. In spite of differences, all three models agree that binding to host specific receptors is a key step in the process involved in cytotoxicity. Several types of receptors for Cry toxins have been reported, such as aminopeptidase N (APN), the cadherin-like proteins, alkaline phosphatases, and ABC transporter [1, 7, 8]. Bt has been used successfully to control many crop pests by transgenic plant or traditional spray approaches, however, many pests are not sensitive to Cry toxins and a number of cases of insect resistance to Cry toxins have been reported as a result of laboratory or field selections [1–3].

Vegetative insecticidal proteins (Vip), which are produced by Bt during its vegetative stages, share no sequence or structural homology with known Cry proteins, and have a wide spectrum of specific insecticidal activity, especially against lepidopteran pests [9]. Vip3 toxins have a different insecticidal process compared with Cry proteins, indicating they are likely to complement and extend the use of Bt insecticidal proteins. A synergistic effect of the toxins in *Spodoptera frugiperda*, *Spodoptera albula*, and *Spodoptera cosmioides* larvae was observed when Vip3Aa and Cry1Ia10 were combined [10]. Moreover, reports showed that transgenic cotton and corn co-expressing Vip3A and Cry1Ab, or Vip3A and Cry1Ac, provided excellent control of target insect species [3, 11–14] and no cross-resistance between Vip3A and Cry proteins was observed [3, 11, 12]. However, compared with Cry toxins, studies on the insecticidal mechanisms of Vip3A are scarce. Lee et al. proposed pore forming as the principal Vip3A mode of action [15]. Our previous work demonstrated that Vip3Aa induces apoptosis in cultured *S*. *frugiperda* (Sf9) cells [16]. Recently, Hernandez-Martinez et al. found that Vip3Aa could induce apoptosis in *Spodoptera exigua* midgut epithelial cells [17]. Reports also showed that Vip3A can not bind to the APN and cadherin-like protein [15]. Instead, it binds to proteins of susceptible insect’s midgut, which are distinct from the known Cry receptors [15, 18]. So far, almost nothing is known on Vip definite receptors except for their molecular weight.

Previously, we and Singh et al. have found Vip3A protoxin has cytotoxicity to *S*. *frugiperda* cells (Sf9 cells and Sf21 cells) [16, 19]. Therefore, we speculated that there are receptors for Vip3Aa in Sf9 cells membrane. In this study, to identify the receptors of Vip3Aa protoxin, we analyzed the Sf9 cells membrane proteins that bind to the biotin labeled Vip3Aa, via the affinity magnetic bead method coupled with nano-HPLC electrospray ion trap mass spectrometry (HPLC-MS/MS). We paid more attention to the scavenger receptor class C like protein (Sf-SR-C) from the 70 identified proteins due to class C scavenger receptors (SR-C) are membrane proteins and have only been identified in insects [20]. We investigated whether Sf-SR-C is the receptor of Vip3Aa both *in vitro* and *ex vivo*. Furthermore, we detected which domain of Sf-SR-C participates in the interaction with Vip3Aa, and validate whether Sf-SR-C mediates the internalization of Vip3Aa, since we observed the presence of Vip3Aa in the cytoplasm of Sf9 cells. Our data confirmed Sf-SR-C acts as the receptor of Vip3Aa, demonstrated the complement control protein (CCP) domain and MAM domain of Sf-SR-C interact with Vip3Aa, and further revealed that endocytosis of Vip3Aa mediated by Sf-SR-C correlates with its insecticidal activity. These results will significantly promote the study and application of Vip3Aa.

## Results

### Vip3Aa can interact with Sf-SR-C *in vitro*

**Fig 1:**
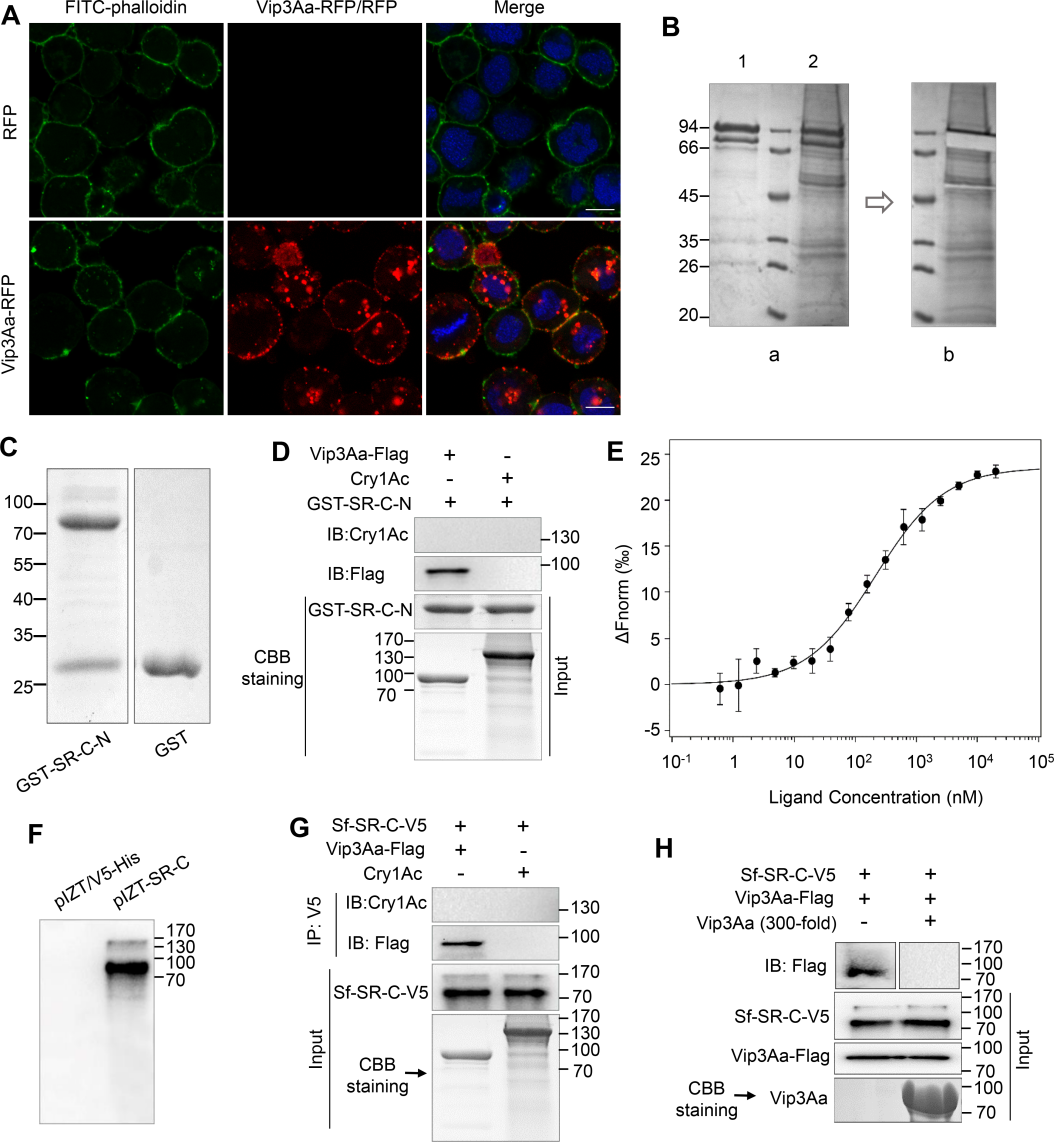
Vip3Aa interacts with Sf-SR-C *in vitr*o. (A) Confocal microscopy images of Sf9 cells treated with RFP or Vip3Aa-RFP (10 μg/mL) for 3 h. Nuclei are stained with DAPI (blue) and cell membrane are stained with FITC-phalloidin (green). Scale bar, 10 μm. (B) Bio-Vip3Aa was incubated with the extracts of Sf9 cell membrane proteins, immunoprecipitated with Streptavidin Mag Sepharose, and detected by Coomassie brilliant blue staining. (a): Lane 1, biotin labeled Vip3Aa; lane 2, the immune complexes. (b): Image of the band of Vip3Aa that was excised from lane 2. (C) The purified GST-Sf-SR-N and GST protein. (D) Vip3Aa-Flag or Cry1Ac were mixed with purified GST-Sf-SR-N, and then the associated complex was pulled down using GST-Sepharose affinity beads followed by immunoblotting (IB) with an anti-Flag antibody or anti-Cry1Ac antibody. (E) MST assay to measure the binding between Vip3Aa and GST-SR-C-N. The labelled Vip3Aa was kept constant at 10 nM, and the GST-SR-C-N is titrated from 0.3 nM to 10 μM. Fitted binding curves and Kd values (mean ± SD) were derived from three independent experiments. (F) Sf9 cells were transiently transfected with the plasmid pIZT-SR-C and the empty vector pIZT/V5-His respectively. 48 h after transfection, cells were collected for immunoblotting with anti-V5 antibody. (G) Sf9-pIZT-SR-C cells lysate was incubated with Vip3Aa-Flag or Cry1Ac, Sf-SR-C in the cells lysate was immunoprecipitated (IP) with anti-V5 antibody, Vip3Aa-Flag and Cry1Ac in the elution was detected by immunoblotting with anti-Flag antibody and anti-Cry1Ac antibody respectively. (H) The lysate of Sf9-pIZT-SR-C cells were subjected to SDS-PAGE, and then transferred to PVDF membranes. The PVDF membranes were probed with Vip3Aa-flag or with Vip3Aa-flag plus unlabeled Vip3Aa without Flag-tag (200-fold), followed by immunoblotting with an anti-Flag antibody.

In addition to the significant virulence effect of Vip3Aa to *S*. *frugiperda* Sf9 cells [16, 19], we also found that Vip3Aa-RFP (a fusion protein of Vip3Aa protoxin and red fluorescence protein) could bind to the Sf9 plasma membrane as shown by colocalization with FITC-phalloidin and internalize in endosomes, while the RFP itself could not (Fig 1A and S1A Fig). Thus, to identify the receptors of Vip3Aa protoxin in Sf9 cells, biotin labeled Vip3Aa (Bio-Vip3Aa) (S1B Fig) was incubated with the extracts of Sf9 cell membrane proteins, immunoprecipitated with Streptavidin Mag Sepharose, and detected by Coomassie brilliant blue staining (Fig 1B, a). The rest of the bands were analyzed by HPLC-MS/MS after the band corresponding to Vip3Aa was excised (Fig 1B, b). Protein sequence database searching of the MS/MS spectra revealed that the bands represented 70 proteins (S1 Dataset), which included 33 ribosomal proteins (in which ribosomal protein S2 had been reported as an interacting partner protein of Vip3A by Singh et al. [19]) and 37 other proteins, including Sf-SR-C (S1C Fig).

At present, class C scavenger receptors (SR-C) have only been identified in insects [20] and only described in *Drosophila melanogaster* [21, 22]. First, the SR-C like gene was cloned from the cDNA of Sf9 cells and named as the *Sf-SR-C* gene (GenBank accession no. KX925839). We then purified the extracellular sequence of Sf-SR-C (aa 20–558) (Sf-SR-C-N) with a glutathione-S-transferase (GST) tag (GST-SR-C-N) (Fig 1C). A GST pulldown assay demonstrated that GST-SR-C-N could bind to Vip3Aa-Flag, but could not bind to the control protein Cry1Ac (Fig 1D). To assess the binding affinity between Vip3Aa protoxin and GST-SR-C-N, we used a microscale thermophoresis assay (MST) in which biomolecular interactions are quantitated by examining the motion of the molecules along a microscopic temperature gradient induced by an infrared laser [23, 24]. The estimated dissociation constant (Kd) was 190 ± 75 nM (Fig 1E). To further test whether full-length Sf-SR-C can interact with Vip3Aa, Sf-SR-C was then transiently expressed in Sf9 cells with a V5 tag after ligation into plasmid pIZT/V5-His (pIZT-SR-C) (Fig 1F). Immunoprecipitation analysis using the anti-V5 antibody showed that Vip3Aa-Flag could be co-immunoprecipitated with Sf-SR-C-V5 (Fig 1G). In the control experiment, we could not detect Cry1Ac after it was incubated with the lysate of Sf9 cells transfected with pIZT-SR-C (Sf9-pIZT-SR-C cells). Ligand blotting was used to detect the specific binding of Sf-SR-C to Vip3Aa. As shown in Fig 1H, Vip3Aa-Flag could bind to Sf-SR-C-V5 and excess Vip3Aa (200-fold) competed for Vip3Aa-Flag binding with Sf-SR-C-V5, which further indicated that Vip3Aa and Sf-SR-C can bind specifically. In addition, via the affinity magnetic bead method, immunoblotting revealed that the Sf-SR-C-V5 from the lysate of Sf9-pIZT-SR-C cells could interact with biotin-labeled Vip3Aa-Flag (S1D Fig). In contrast, Sf-SR-C-V5 could not interact with control biotin labeled ChiB-flag (Chitinase B secreted by Bt). These results indicated Vip3Aa protoxin can interact with Sf-SR-C *in vitro*.

### Sf-SR-C acts as the receptor of Vip3Aa *ex vivo*

**Fig 2:**
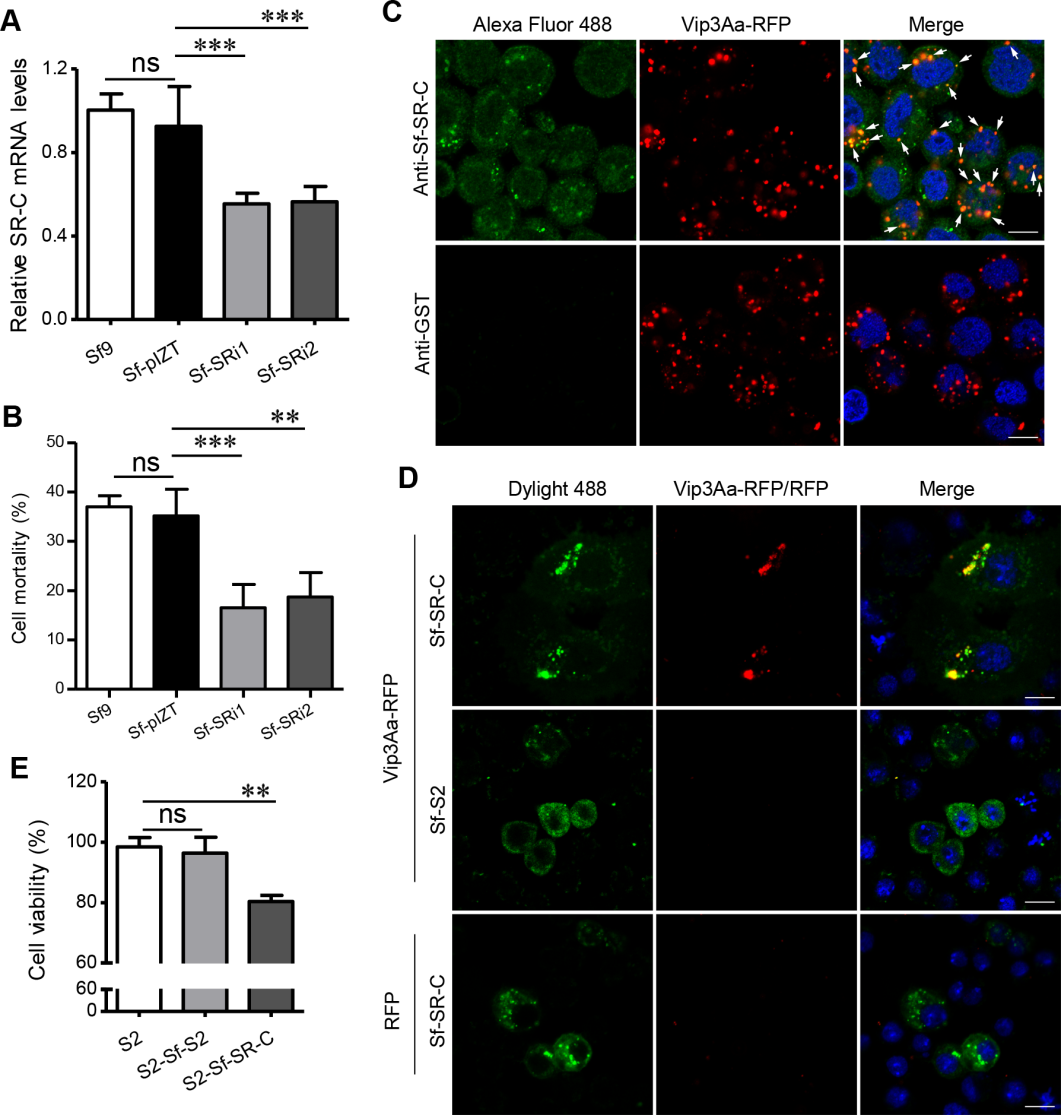
Sf-SR-C acts as the receptor for Vip3Aa *ex vivo*. (A) qRT-PCR analysis of the relative transcript levels of *SR* genes in the cell lines of Sf9, Sf-pIZT, Sf-SRi1 and Sf-SRi2; Data are expressed as the mean ± SD from three independent experiments; ns, non-significant; *** P < 0.001; one-way ANOVA with Dunnett’s method. (B) Cell mortality of different cell lines (Sf9, Sf-pIZT, Sf-SRi1 and Sf-SRi2) exposed to 50 μg/mL of Vip3Aa for 48 h. Data are expressed as the mean ± SD from three independent experiments; ns, non-significant; ** P < 0.01; one-way ANOVA with Duncan method. (C) Confocal microscopy sections showing the localization of Vip3Aa-RFP (red) and Sf-SR-C (green) in Sf9 cells. The anti-Sf-SR-C-N polyclonal antibody and Alexa Fluor 488-conjugated anti-rabbit antibody were used to show the location of Sf-SR-C in Sf9 cells. Anti-GST polyclonal antibodies were used as the control. Arrowheads point to co-localization between Vip3Aa-RFP and Sf-SR-C. Nuclei are stained with DAPI (blue). Scale bar, 10 μm. (D) S2 cells were transfected with Sf-SR-C or Sf-S2. 48 h after transfection, cells were exposed to Vip3Aa-RFP or RFP (red), fixed, and then immunostained with Dylight 488-conjugated anti-V5 antibodies (green). Nuclei are stained with DAPI (blue). Scale bar, 10 μm. (E) Cell viability of different S2 cell lines (S2, S2-Sf-S2 and S2-Sf-SR-C) exposed to 25 μg/mL of Vip3Aa for 48 h (The transfection efficiency was about 30%). Data are expressed as the mean ± SD from three independent experiments; ns, non-significant; ** P < 0.01; one-way ANOVA with Duncan method.

To verify the role of Sf-SR-C in Vip3Aa protoxin binding to Sf9 cells in more detail, we attempted to generate Sf9 cells in which the expression of endogenous *Sf-SR-C* gene was reduced. Two plasmids, pIZT-SRi1 and pIZT-SRi2, which can generate fragments of double-stranded RNA (dsRNA) from the *Sf-SR-C* gene (S2A and S2B Fig) [25], were stably transfected into Sf9 cells, respectively, which resulted in the generation of Sf-SRi1 and Sf-SRi2 cell lines. As the quantitative real-time reverse transcription PCR (qRT-PCR) result shown in Fig 2A; the expression level of the *Sf-SR-C* gene was markedly reduced in the Sf-SRi1 and Sf-SRi2 cells compared with the Sf9 cells and the cells stably transfected with pIZT/V5-His (Sf-pIZT cells). Consistent with this, a CCK-8 cytotoxicity assay results showed that the cytotoxic effects of Vip3Aa on the Sf-SRi1 and Sf-SRi2 cells were also clearly reduced compared with those on Sf9 cells and Sf-pIZT cells (Fig 2B).

Next, we carried out co-localization assays to detect the interaction between Vip3Aa and Sf-SR-C. After treating the Sf9 cells with Vip3Aa-RFP for 6 h, we monitored the Vip3Aa and Sf-SR-C distribution using immunofluorescent staining. The anti-Sf-SR-C-N polyclonal antibody and Alexa Fluor 488-conjugated anti-rabbit antibody were used to show the location of Sf-SR-C in Sf9 cells. As shown in Fig 2C, most of the dots of Vip3Aa-RFP were co-located with Sf-SR-C, especially in the dots that were Sf-SR-C-rich. In the control experiment that the anti-GST polyclonal antibody was used, we detected almost no green fluorescence.

We also observed that Vip3Aa-RFP has almost no affinity for *Drosophila* S2 cells (S2 cells) (S3A Fig). We therefore cloned the gene of *Sf-SR-C* into plasmid pAc5.1/V5-HisB (pAc-Sf-SR-C) and transiently transfected it into S2 cells (S2-Sf-SR-C cells) to examine the specific interaction of Vip3Aa and Sf-SR-C in S2 cells. The ribosomal S2 protein of Sf9 cells (Sf-S2) was also heterologously expressed into the S2 cells (S2-Sf-S2 cells) as a control (S3B Fig). The Dylight 488 conjugated anti-V5 antibody was used to show the heterologously expressed protein in S2 cells. After treating the S2 cells with Vip3Aa-RFP for 12 h, immunofluorescent staining showed that Vip3Aa-RFP could clearly bind to the S2-Sf-SR-C cells, and the dots of Vip3Aa-RFP were co-located with the dots that were rich in Sf-SR-C (Fig 2D and S3C Fig), similar to the phenomenon that Vip3Aa-RFP binds to Sf-SR-C in Sf9 cells. In contrast, we didn’t detect the interaction between Vip3Aa-RFP and S2-Sf-S2 cells, nor did we find the binding of RFP to S2-Sf-SR-C cells. In addition, the cytotoxicity assay showed Vip3Aa protoxin is more toxic to S2-Sf-SR-C cells than to S2-Sf-S2 and S2 cells (Fig 2E) (The transfection efficiency was about 30%). Taken together, these results revealed that Sf-SR-C could also interact with Vip3Aa protoxin *ex vivo*.

### Vip3Aa interacts with Sf-SR-C to trigger the death of target insects

**Fig 3:**
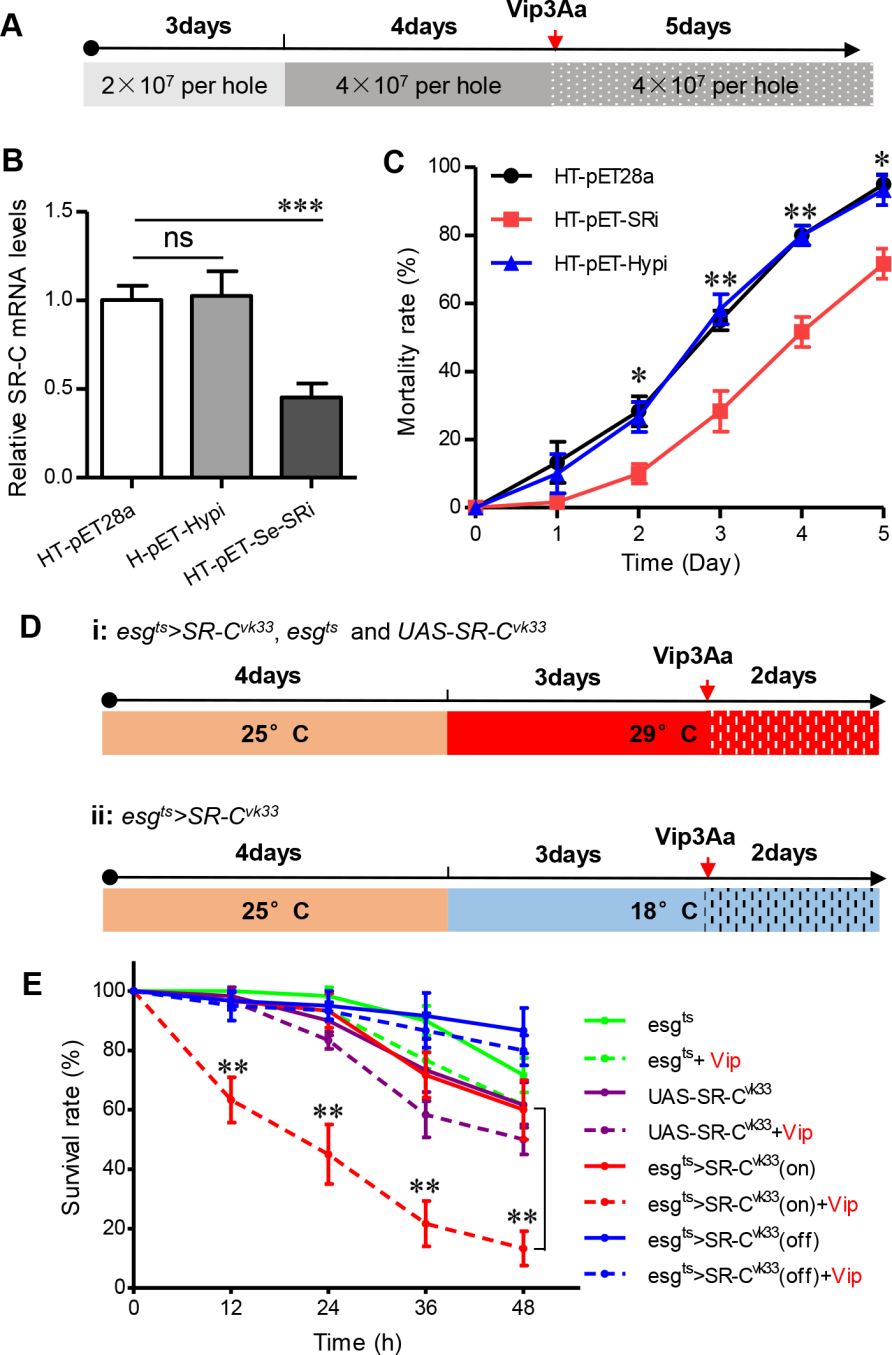
Bioassay to validate Sf-SR-C as the receptor for Vip3Aa. (A) Scheme for the *S*. *exigua* bioassays. The 1^st^ instar *S*. *exigua* larvae were first fed with the HT-pET-Se-SRi, HT-pET-Hypi and HT-pET28a strains for 7 d, respectively (For the first three days, the number of bacteria in each hole was 2 × 10^7^; for the next four days, the number of bacteria in each hole was 4 × 10^7^ per well.). And then 3^st^ instar larvae were selected from each group to detect the transcription level of the *Se-SR-C* gene in the midgut of the larvae. The larvae were then exposed to Vip3Aa (0.8 μg/cm^2^) and the strains (4 × 10^7^ per well) respectively for another 5 d to detect the mortality rate. (B) qRT-PCR analysis of the relative transcript levels of the *SR-C* gene in the midgut of *S*. *exigua* larvae after feeding with the HT-pET-Se-SRi, HT-pET-Hypi and HT-pET28a strains for 7 d, respectively. Data are expressed as the mean ± SD from three independent experiments; ns, non-significant; *** P < 0.001 by two-tailed Student’s t tests compared with the corresponding control value. (C) Mortality rate of *S*.*exigua* larvae feed with the bacterially-expressed dsRNA (feeding with HT-pET-Se-SRi, HT-pET-Hypi and HT-pET28a, respectively) after exposure to Vip3Aa (0.8 μg/cm^2^) (n = 20). The survival rates of each group were analyzed every day. Data are expressed as the mean ± SEM from three independent experiments; ns, non-significant; * P < 0.05, ** P < 0.01 by two-tailed Student’s t tests compared with the corresponding control value. (D) Scheme for the *Drosophila* bioassays. The adult flies of different strains were transferred to fresh medium and reared at 25 °C for 4 days. Then the flies strains (adult flies and their larvae) were shifted to 29 °C (Gal4 ‘‘on”) or 18 °C (Gal4 ‘‘off”) for 3 days. The 2^st^ instar larvae of each strains were picked separately and treated with Vip3Aa or dialysis buffer for 48 h. The survival rates of each group were analyzed every 12 h. (E) Survival rate of different fly strains treated with Vip3Aa (100 μg/mL) or dialysis buffer (n = 20). The survival rates of each group were analyzed every 12 h. Data are expressed as the mean ± SD from three independent experiments; ** P < 0.01 by two-tailed Student’s t tests compared with the corresponding control value.

Vip3Aa has a high affinity for IOZCAS-Spex-II-A cells (*Spodoptera exigua* cells) (S3A Fig) and shows a significant toxic effect to *S*. *exigua* [10]. We also cloned two partial sequences with similarity to the *Sf-SR-C* gene from the total cDNA of *S*. *exigua* cells (GenBank accession no. KY829113 and MF969248). Therefore, we attempted to use ingestion of bacterially expressed dsRNA to reduce the expression of the *S*. *exigua* larvae midgut *SR-C* gene (Se-SR-C) to detect whether it affected the toxicity of Vip3Aa to the larvae. The vector pET-Se-SRi, which expresses a partial dsRNA of the *Se-SR-C* gene (S2C Fig), was transformed into bacterial strain HT115 (DE3), which lacks RNase III activity to express dsRNA fragment of Se-SR-C (HT-pET-Se-SRi) [26] (S4A Fig). The vector pET-Hypi, which expresses a partial dsRNA of a hypothetical protein (Hyp) (GenBank: PCG66164.1), and the blank plasmid pET28a were transformed into the HT115 strain as control (HT-pET-Hypi and HT-pET28a). The qRT-PCR results showed that after continuous feeding of the *S*. *exigua* larvae with the strains for 7 days (Fig 3A and S4B Fig), the transcription level of the *Se-SR-C* gene of the larvae fed with the HT-pET-Se-SRi strain was effectively inhibited compared with the control (Fig 3B). The larvae were then exposed to Vip3Aa and the strains for another 5 d to detect the survival rate. The bioassay results shown in Fig 3C indicated that the mortality rate of the larvae in which the *Se-SR-C* gene was silenced was clearly lower than that of the control, which suggested that reducing the expression of the *Se-SR-C* gene in *S*. *exigua* lavae decrease their sensitivity to Vip3Aa.

Vip3A has high insecticidal activity against Lepidopteran rather than Dipteran [9]. To further examine the interaction of Sf-SR-C with Vip3Aa in an insect that is insensitive to Vip3Aa (S4C Fig), we constructed transgenic *Drosophila* that overexpressed Sf-SR-C using the *esg-Gal4 tub-Gal80*^*ts*^ system. In this system, the *esg-Gal4* driver is mainly active in the midgut cells of *Drosophila* and Gal4 is under the control of a temperature sensitive Gal80 that allows the conditional induction of the UAS-linked *Sf-SR-C* gene [27]. After culturing at 25 °C for 4 d, the fly strains were shifted to 29 °C (Gal4 ‘‘on”) or 18 °C (Gal4 ‘‘off”) for 3 d (Fig 3D). The about 2-day-old larvae were then treated with Vip3Aa or dialysis buffer for 48 h and the survival rates were detected (Fig 3D and S4D Fig). As shown in Fig 3E, the *Drosophila* larvae that overexpressed Sf-SR-C in their midgut (*esg*^*ts*^*>SR-C*^*vk33*^) (Red) had a significantly higher mortality rate after exposure to Vip3Aa compared with the control group, which was treated with dialysis buffer. In the group of *esg*^*ts*^ (Green) and *UAS-SR-C*^*vk33*^ (Purple), which could not express Sf-SR-C, Vip3Aa showed no obvious toxicity to the larvae compared with the control. Moreover, shutdown of the expression of Sf-SR-C in the gut epithelia of the larvae (18 °C treated) eliminated the toxicity of Vip3Aa to the larvae (Blue). These results further indicated Sf-SR-C is the receptor for Vip3Aa, which causes the death of sensitive insects.

### Vip3Aa bind to the MAM and CCP domains of Sf-SR-C

**Fig 4:**
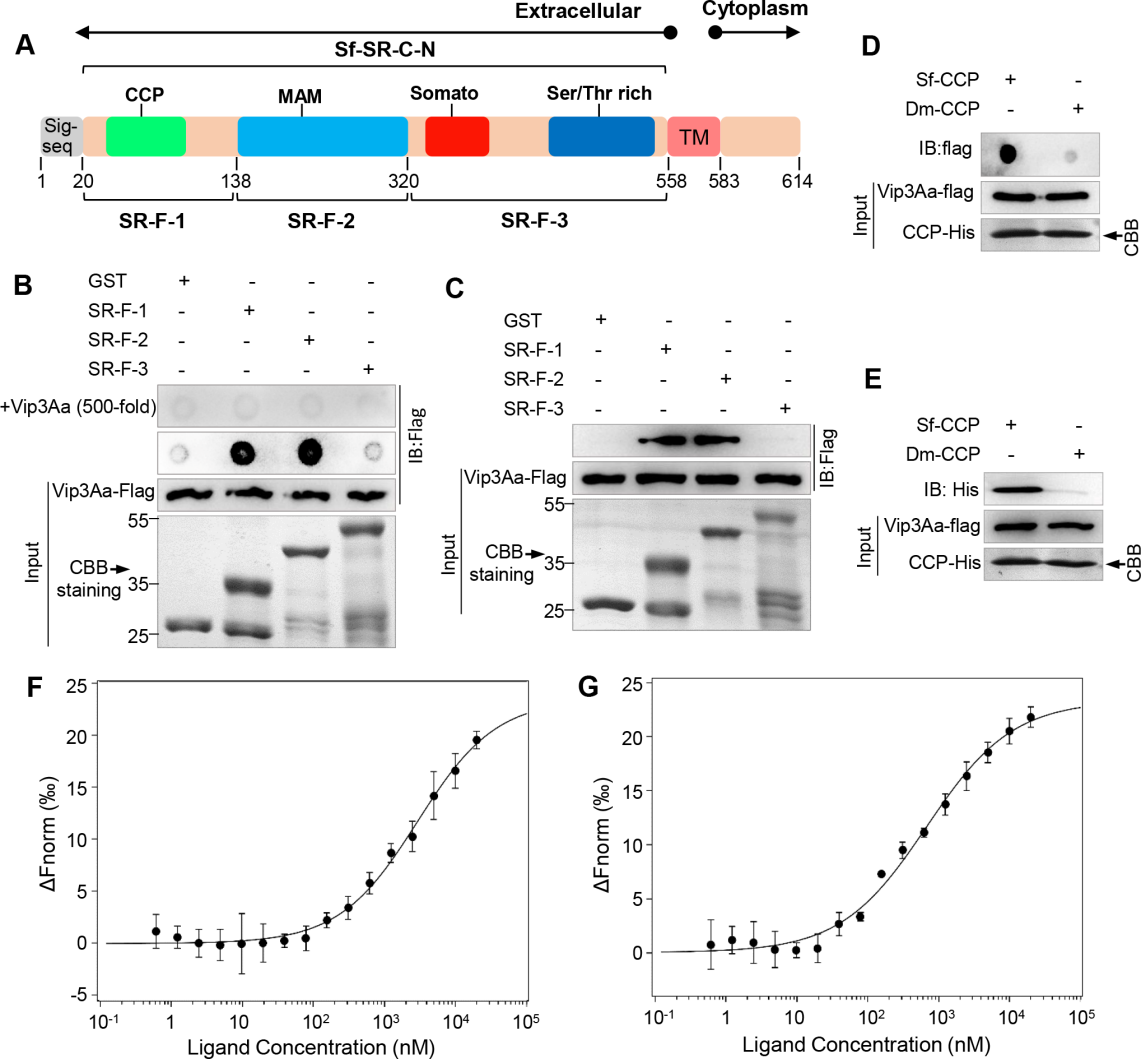
Vip3Aa binds to MAM and CCP domains. (A) Schematic representation of Sf-SR-C, indicating the different domains. Complement control protein (CCP), MAM, somatomedin B (somato), and Ser/Thr rich domains. Sig-seq: signal peptide sequence, TM: transmembrane. The numbers represent the number of amino acids at the position. (B) GST, GST-SR-C-F-1, GST-SR-C-F-2, and GST-SR-C-F-3 proteins were dotted on a PVDF membrane directly and were then probed with Vip3Aa-flag or with Vip3Aa-flag plus unlabeled Vip3Aa without Flag-tag (500-fold), followed by immunoblotting with an anti-Flag antibody. (C) GST-SR-C-F-1, GST-SR-C-F-2, and GST-SR-C-F-3 conjugated to GST-Sepharose affinity beads respectively, and then incubated with Vip3Aa-flag. GST was used as the control and immunoblotting used the anti-Flag antibody. (D) Dot blotting to detect the interaction of Sf-CCP with Vip3Aa-flag; Dm-CCP was used as the control and immunoblotting used the anti-Flag antibody. (E) Pulldown experiments: Vip3Aa-flag conjugated to protein G agarose beads using an anti-flag antibody, and incubated with Sf-CCP-His or Dm-CCP-His, followed by immunoblotting with an anti-His antibody. (F) and (G) The binding affinity of Vip3Aa with CCP (F) and MAM (G) domains of Sf-SR-C were analysed with MST. The labelled Vip3Aa was kept constant at 10 nM, and the CCP and MAM domains are titrated from 0.3 nM to 10 μM, respectively. Fitted binding curves and Kd values (mean ± SD) were derived from three independent experiments.

From BLASTP analysis, we found that the protein sequence of Sf-SR-C was not similar to the SR-C from *D*. *melanogaster* (dSR-CI) (only about 27% sequence identity). However, the extracellular sequence of Sf-SR-C has four structural domains that are similar to dSR-CI, including the CCP, MAM, somatomedin B, and Ser/Thr rich domains (Fig 4A). To further investigate which domain of Sf-SR-C mainly participates in the interaction with Vip3Aa protoxin, the extracellular sequence of Sf-SR-C was divided into three parts (SR-F-1, SR-F-2, and SR-F-3) (Fig 4A) and expressed as fusion proteins with GST. Dot blotting analysis revealed that GST-SR-F-1 (aa 20–138), which contains the CCP domain (aa 26–76), and GST-SR-F-2 (aa 139–320), which is the MAM domain, could bind to Vip3Aa-Flag, while GST and GST-SR-F-3 (aa 321–558) could not (Fig 4B). Furthermore, excess Vip3Aa (500-fold) competed for Vip3Aa-Flag binding with GST-SR-F-1 and GST-SR-F-2 (Fig 4B). Moreover, pulldown experiments also revealed that GST-SR-F-1 and GST-SR-F-2 could directly interact with Vip3Aa-Flag (Fig 4C). These results indicated that the binding of SR-F-1 and SR-F-2 with Vip3Aa-Flag was specific. GST-SR-F-1 contained regions other than the CCP domain; therefore, we further purified Sf-CCP (CCP domain of Sf-SR-C (aa 20–80) with a His-tag) to detect the interaction with Vip3Aa-Flag, and the Dm-CCP (CCP domain (aa 20–80) of dSR-CI (GenBank: U17693.1)) was used as a control. Both dot blotting analysis (Fig 4D) and pulldown assays (Fig 4E) verified the physical interaction between Sf-CCP and Vip3Aa-Flag. The results also showed that the Dm-CCP domain could not bind to Vip3Aa-Flag, which further validated the specific binding between Vip3Aa and Sf-SR-C. Furthermore, MST was also applied to assay the binding affinity of Vip3Aa protoxin with Sf-CCP and MAM domains (Fig 4F and 4G). The determined Kd values were 2.19 ± 1.55 μM and 463 ± 117 nM, respectively. These results certified the CCP and MAM domains of Sf-SR-C could bind to Vip3Aa protoxin.

### Vip3Aa enter into Sf9 cells via endocytosis

**Fig 5:**
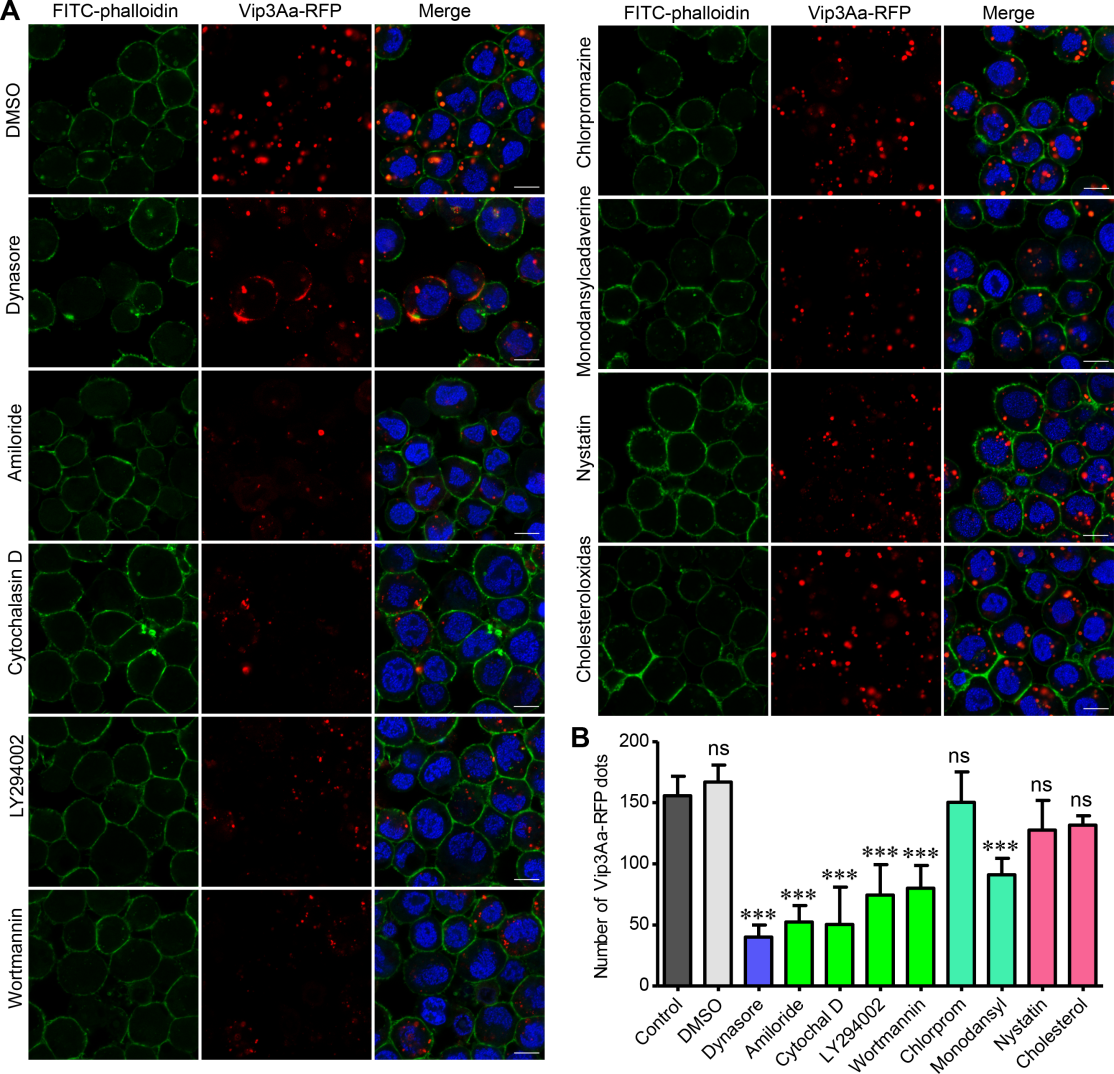
Vip3Aa entry into Sf9 cells via endocytosis. (A) Confocal images showing Vip3Aa-RFP dots in Sf9 cells. Cells were pre-treated with different inhibitors for 1 h and then co-incubated with Vip3Aa-RFP (4.5μg/ml) for 6 h. Nuclei are stained with DAPI (blue) and cell membrane are stained with FITC-phalloidin (green). Scale bar, 10 μm. (B) Quantification of the number of conspicuous Vip3Aa-RFP dots in Sf9 cells of (A) in a blind fashion (n = 60 cells per sample). Cytochal D: Cytochalasin D, Chlorprom: Chlorpromazine, Monodansyl: Monodansylcadaverine, Cholesterol: Cholesteroloxidas. Data are expressed as the mean ± SD from three independent experiments; ns, non-significant; *** P < 0.001; one-way ANOVA using Duncan method.

As Figs 1A and 2C showed above, we observed red dots in the cytoplasm of Sf9 cells after exposing them to Vip3Aa-RFP, which suggested the internalization of Vip3Aa. We first used several inhibitors of endocytosis to test whether Vip3Aa-RFP could enter the Sf9 cells via endocytosis [28, 29]. As shown in Fig 5A and 5B, dynasore, which is an inhibitor of dynamin, could significantly inhibit Vip3Aa-RFP entry into Sf9 cells. The known macropinocytosis inhibitors, amiloride, cytochalasin D, LY294002, and wortmannin also reduce the number of red dots inside the Sf9 cells. However, among two inhibitors of clathrin-mediated endocytosis (chlorpromazine and monodansylcadaverine) and two inhibitors of clathrin-independent endocytosis (nystatin and cholesterol-oxidase), only monodansylcadaverine could reduce the number of red dots in Sf9 cells; the others had no effect on the number of Vip3Aa-RFP dots in the Sf9 cells compared with the control. These results suggested Vip3Aa enter Sf9 cells through dynamin-dependent and macropinocytosis-related endocytosis.

### Sf-SR-C mediates the internalization of Vip3Aa

**Fig 6:**
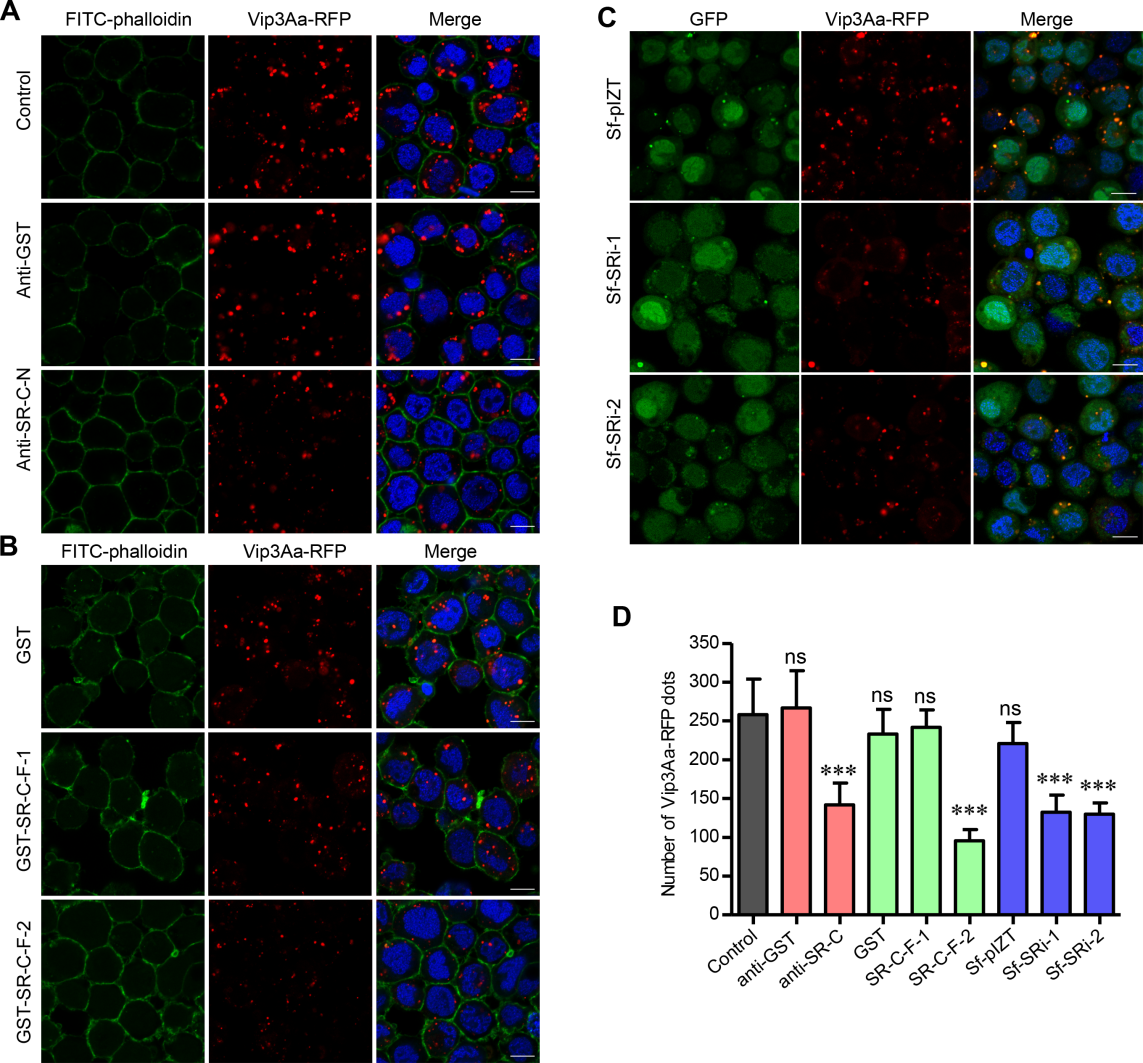
Sf-SR-C mediates the internalization of Vip3Aa. (A) Confocal microscopy sections showing Vip3Aa-RFP dots in the Sf9 cells. The cells were separately incubated with purified anti-Sf-SR-C-N polyclonal antibodies and anti-GST polyclonal antibodies for 1 h and the cells were then co-incubated with Vip3Aa-RFP (4.5μg/ml) for another 6 h. Nuclei are stained with DAPI (blue) and cell membrane are stained with FITC-phalloidin (green). Scale bar, 10 μm. (B) Confocal images showing Vip3Aa-RFP dots in the Sf9 cells. The cells were treated by Vip3Aa-RFP (4.5μg/ml) combined with an excess of GST-SR-F-1 and GST-SR-F-2 (20-fold) respectively. Nuclei are stained with DAPI (blue) and cell membrane are stained with FITC-phalloidin (green). Scale bar, 10 μm. (C) Confocal images showing Vip3Aa-RFP dots in the Sf-pIZT, Sf-SRi1 and Sf-SRi1 cells after incubating with Vip3Aa-RFP (4.5μg/ml) for 6 h. Nuclei are stained with DAPI (blue). Scale bar, 10 μm. (D) Quantification of the number of conspicuous Vip3Aa-RFP dots in Sf9 cells of (A), (B) and (C) in a blind fashion (n = 100 cells per sample). Data are expressed as the mean ± SD from three independent experiments; ns, non-significant; *** P < 0.001; one-way ANOVA using Duncan method.

One of the main functions of scavenger receptors (SRs) is endocytosis [20]. Thus, we hypothesized that Vip3Aa enters the Sf9 cells via endocytosis mediated by Sf-SR-C. To further verify Sf-SR-C mediated the internalization of Vip3Aa, purified anti-Sf-SR-C-N polyclonal antibodies were incubated with the Sf9 cells for 1 h and the cells were then co-incubated with Vip3Aa-RFP for another 6 h. The results showed that the number of red dots in the cytoplasm of Sf9 cells was reduced visibly after treatment with the anti-Sf-SR-C-N polyclonal antibody, while there are many red dots in cells treated with anti-GST polyclonal antibodies (Fig 6A and 6D). We also quantified the number of red dots in the Sf-SRi1 and Sf-SRi2 cell lines. Compared with the Sf-pIZT cells, the internalization of Vip3Aa-RFP also reduced markedly in the Sf-SRi1 and Sf-SRi2 cells (Fig 6C and 6D). Furthermore, because we found that Vip3Aa can bind to GST-SR-F-1 and GST-SR-F-2, Vip3Aa-RFP combined with an excess of GST-SR-F-1 and GST-SR-F-2 (20-fold) were exposed to Sf9 cells, respectively. The competitive binding assay showed the amount of red dots in the Sf9 cells treated by Vip3Aa-RFP and GST-SR-F-2 was significantly decreased compared with the control cells treated with Vip3Aa-RFP and GST (Fig 6B and 6D). However, in the case of GST-SR-F-1, such phenomenon did not occur, which suggested the MAM domain might play more critical role in the internalization of Vip3Aa than the CCP domain. These results indicated the Sf-SR-C mediates the internalization of Vip3Aa via endocytosis.

### Endocytosis of Vip3Aa correlates with its insecticidal activity

**Fig 7:**
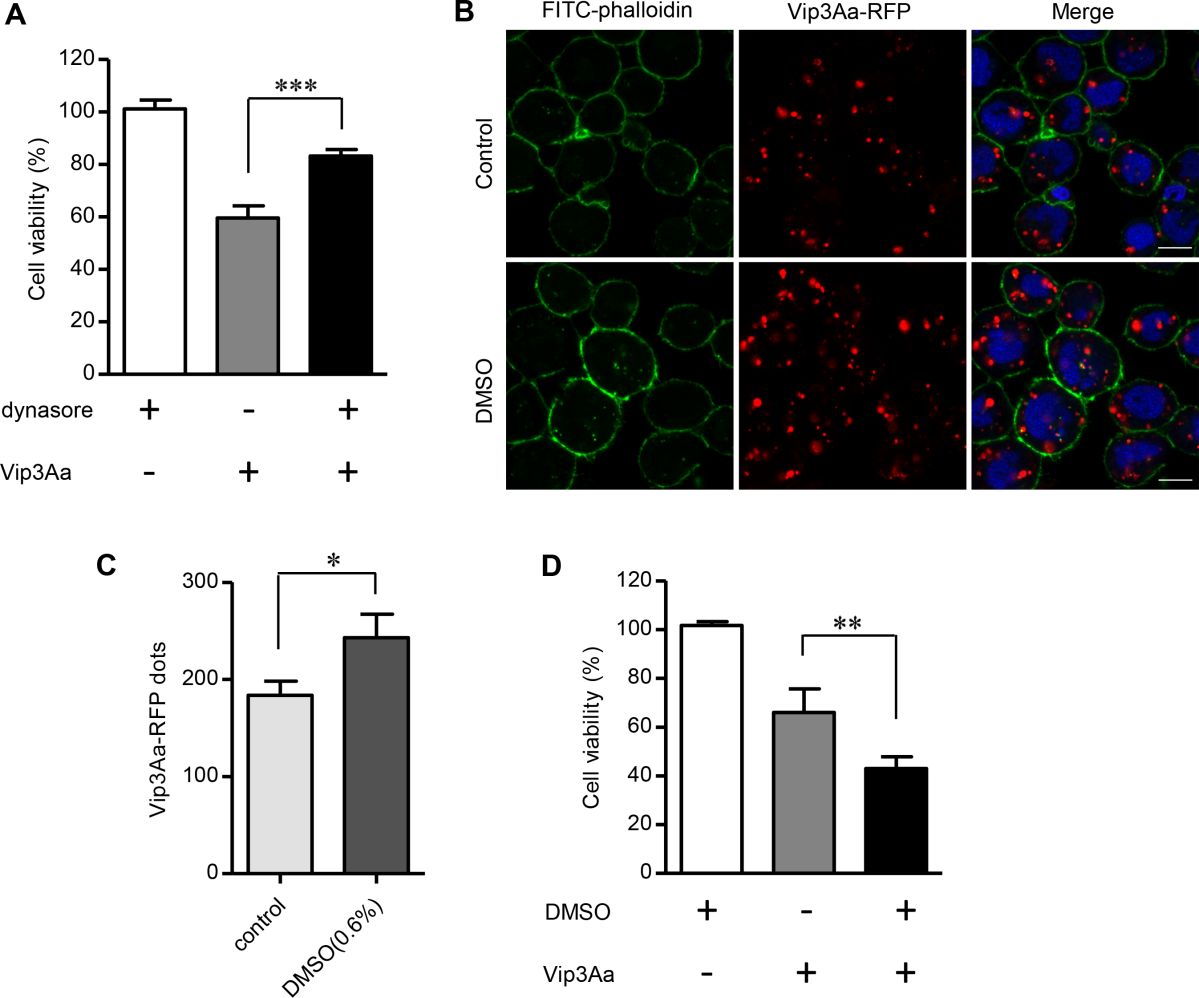
Vip3Aa entering cells is directly related to its toxicity. (A) Cell viability of Sf9 cells treated with or without Vip3Aa (50 μg/mL) for 48 h in the presence or absence of the dynasore (4μM). (B) Confocal microscopy sections showing Vip3Aa-RFP dots in the Sf9 cells. The cells were treated by Vip3Aa-RFP (2.5 μg/mL) for 6 h in the presence or absence of the DMSO (0.6%). Nuclei are stained with DAPI (blue) and cell membrane are stained with FITC-phalloidin (green). Scale bar, 10 μm. (C) Quantification of the number of conspicuous Vip3Aa-RFP dots in Sf9 cells of (B) in a blind fashion (n = 100 cells per sample). (D) Cell viability of Sf9 cells treated with or without Vip3Aa (50 μg/mL) for 48 h in the presence or absence of the DMSO (0.6%). (A), (C) and (D): data are expressed as the mean ± SD from three independent experiments; * P < 0.05, ** P < 0.01, *** P < 0.001 by two-tailed Student’s t tests compared with the corresponding control value.

The above results showed that silencing of *Sf-SR-C* gene could clearly reduce the toxicity of Vip3Aa to Sf9 cells (Fig 2B) and also reduce the amount of Vip3Aa entering into Sf9 cells (Fig 6B and 6D), which suggested the amount of Vip3Aa entering cells is directly related to its toxicity. Therefore, we carried out further experiments to verify this speculation. Firstly, we have demonstrated that the endocytosis inhibitor dynasore could significantly inhibit the internalization of Vip3Aa, without affecting the binding of Vip3Aa to Sf9 cells (Fig 5A and 5B and S5 Fig). Through cytotoxicity assay (Fig 7A), we further found that dynasore (4μM) markedly decreased the toxicity of Vip3Aa to Sf9 cells while reducing the entry of Vip3Aa into cells. Dynasore alone did not affect the survival of Sf9 cells. In addition, in the Fig 5A and 5B, we found that the DMSO (0.1%) had a tendency to increase the number of Vip3Aa into Sf9 cells. So we explored the highest concentration of DMSO that did not cause toxicity to Sf9 cells. As shown in Fig 7B and 7C, we found that when the concentration of DMSO increased to 0.6% (v/v), it could clearly increase the number of Vip3Aa entering Sf9 cells. Moreover, the cytotoxicity assay also showed that DMSO increased Vip3Aa's toxicity to Sf9 cells while promote the internalization of Vip3Aa (Fig 7D), and DMSO alone had no obvious toxicity to Sf9 cells. These results further demonstrated that the internalization of Vip3Aa is directly related to its toxicity. Taken together, our results indicated the induced mortality of Vip3Aa in Sf9 cells correlated with its endocytosis mediated by Sf-SR-C.

## Discussion

Vip3Aa proteins have been studied for more than 20 years since they were first found by Estruch et al. in 1996 [30]. They are considered as novel insecticidal toxins secreted by Bt because they have different insecticidal properties compared with known Cry toxins and have a broad insecticidal spectrum within Lepidoptera [9]. To date, more than 138 Vip proteins have been found and were divided into four categories according to the classification of Bt Toxin Nomenclature Committee [31]. However, there has been no report of a definite receptor for Vip toxins up to now. In this paper, via HPLC-MS/MS, 70 potential binding proteins of Vip3Aa, including ribosomal protein S2 and actin, were identified (S1 Dataset). Singh et al. identified ribosomal protein S2 as a toxicity-mediating interacting partner protein of Vip3A in Sf21 cells [19]. However, as an intracellular protein, S2 protein is not likely to be a receptor of Vip3A. That maybe why Singh et al named it interacting partner protein, not a receptor. Our results also showed Vip3Aa could not bind to the S2-Sf-S2 cells, which heterologously expressed Sf-S2 into the S2 cells, and had no obvious cytotoxicity to them (Fig 2D and 2E). It suggests that S2 protein is not a receptor for Vip3Aa. Actin was identified as a novel Cry1Ac binding protein in *Manduca sexta* midgut through proteomic analysis [32]. For the same reason, it is unlikely that this protein is serving as a receptor for Vip3Aa. We speculate that the Vip3Aa may interfere with the function of the ribosome and actin after entering the cells.

Estruch et al. mentioned that a 48-kDa protein from *Agrotis ipsilon* with homology to tenascins may act as the receptor of Vip3A in their patent [33]. However, they did not provide any supporting data and no subsequent reports proved their speculation. Furthermore, consistent with previous reports [9], we did not find the receptors for Cry toxins such as APN or cadherin-like proteins in the 70 proteins we identified, which suggested Vip3Aa share no binding sites with Cry toxins. Moreover, we also demonstrated that Cry1Ac could not bind to Sf-SR-C (Fig 1D and 1G). These results further strengthen the viewpoint that Vip3 toxins and Cry toxins have different mechanisms of action, which makes Vip3 toxins good candidates for combination with Cry toxins in transgenic plants to prevent or delay insect resistance and to broaden the insecticidal spectrum.

In this study, we provide *in vitro*, *ex vivo*, and bioassay evidences for the first time confirming that the SR-C-like protein Sf-SR-C from Sf9 cells is the receptor for Vip3Aa. Scavenger receptors are cell surface receptors that typically bind multiple ligands and promote the removal of non-self or altered-self targets. SRs are classified into 10 classes [20]. At present, the vast majority of SRs have been identified and studied in mammals; however, SR-C have only been found in insects and have only been described in *Drosophila* [21, 22]. In mammalian cells, SRs play a crucial role in maintenance of host homeostasis, and also participate in host immune responses and metabolism. They can recognize and bind to a broad spectrum of ligands, including modified and unmodified host-derived molecules or microbial components [21, 34]. However, researchers also found that pathogens have evolved mechanisms to subvert SRs’ function to infect host cells [34]. For example, hepatitis C virus [35], enterovirus 71 (EV71) [36], and coxsackievirus (CVA7, CVA14 and CVA16) [37] utilize class B receptors to infect host cells. *Chlamydia trachomatis* uses the lipid transfer activity of SR-B1 for survival in host cells [38]. Even the class B scavenger receptor CD36, which has been implicated in the clearance of several bacterial and protozoan pathogens, has been reported to be co-opted by mycobacteria [39]. In *D*. *melanogaster*, SR-CI was identified as the recognition receptor for acetylated low-density lipoprotein [22] and bacteria [40]. In addition, Philips et al. found that Peste in *D*. *melanogaster* (a CD36 homolog) is involved in the uptake of mycobacteria into host cells [41]. In this study, we provide another example, in which the bacterial toxin Vip3Aa can exploit Sf-SR-C of Sf9 cells to kill host cells.

In addition, the Vip1 and Vip2 proteins which were first found in *Bacillus cereus* are regarded as binary toxins. Vip1 is speculated as the binding component and triggers endocytosis, and Vip2 enters the cell and exerts its toxic effect [9]. Vip3Aa has no sequences similarity to Vip1 or Vip2, however, our results certified Vip3Aa can entry into the Sf9 cells by itself via the endocytosis mediated by Sf-SR-C.

In insects, there has been little research into the mode of action of SR-C. However, in mammals, one of the main functions of SR proteins is endocytosis, which can trigger a series of signaling pathways [21, 34]. More interestingly, SR function is increasingly linked to apoptosis in a wide variety of cell types. Binding of fucoidan ligand by the macrophage SR-A1 triggers endocytosis by caveolae-dependent pathways, which stimulates apoptosis via a p38 MAPK and JNK dependent intracellular signaling pathway [42]. In vascular cells, thrombospondin-1 activation of SR-B2 triggers downstream signaling through p38 MAPK and caspase dependent pathways with increased apoptosis [43]. In addition, SR-E1 function is linked to apoptosis in the endothelium, vascular smooth muscle cells, macrophages, epithelial cells and neurons. [44,45]. As mentioned above, some pathogens can utilize the function of SRs to invade the host cell. Some toxins secreted by bacteria can also entry into host cell via endocytosis to exert their toxic effects. Diphtheria toxin, an exotoxin secreted by *Corynebacterium diphtheriae* and causes the disease diphtheria in humans, is believed to enter toxin-sensitive mammalian cells by receptor-mediated endocytosis and inhibit protein synthesis of host cells [46, 47]. In this way, it acts as a RNA translational inhibitor and results into cell apoptosis. Receptor-mediated endocytosis is required for efficient expression of toxicity. Once endocytosis was inhibited, the cytotoxicity of diphtheria toxin was blocked accordingly [46, 47]. Our results indicated that the toxicity of Vip3Aa to Sf9 cell correlated with its endocytosis mediated by Sf-SR-C (Figs 5, 6 and 7). It suggested that internalization is essential for Vip3Aa to exert its toxic effects.

Endocytosis is mentioned in Cry5- *Caenorhabditis elegans* system. In that case, however, endocytosis is a protection strategy utilized by worms to against the toxin Cry5 [48]. As to the “signal transduction” model, endocytosis is not an indispensable step [4]. In newly found “necrosis” model, Cry6A toxin is also internalized into intestinal cells, but cell death induced by Cry6Aa does not depend on the apoptotic mechanism [6]. These further implied the mode of action of Vip3Aa toxins different from that of Cry toxins. However, the more detailed mechanisms of how Vip3Aa kills Sf9 cells after interacting with Sf-SR-C and the follow-up connection with our previous results that Vip3Aa can induce apoptosis in Sf9 cells [16], are complex and interesting and will require further study.

In cytotoxicity assays, we used Vip3Aa protoxin. However, we found that the purified Vip3Aa protoxin was unstable. As shown in S1B Fig, in the biotin labeled Vip3Aa (lane 3), we can see the emergence of the activated Vip3Aa like protein (about 66 kDa). After incubating Vip3Aa protoxin with Sf9 cells, western blotting revealed that the activated Vip3Aa like protein was also apparent in the medium (S6 Fig). Therefore, it is difficult to exclude the existence of activated Vip3Aa in the process of toxicity testing. Lee et al. have already demonstrated that the activated Vip3Aa has the pore formation activity. In contrast, the full-length Vip3Aa protein was unable to form pores [15]. They proposed formation of ion channels as the principal mode of action of activated Vip3Aa. However, in this work, we have demonstrated that full-length Vip3Aa could bind to the Sf-SR-C receptor and endocytosis of Vip3Aa correlates with its toxicity.

In addition, the activated Vip3Aa protein was considered to correspond to the C terminus of the Vip3Aa protoxin (from amino acid 199 to the end) [9, 33]. We also purified the activated Vip3Aa protein (Vip3Aa-199) (S7A Fig). Through cytotoxicity assay, we found that although the activated Vip3Aa is also toxic to Sf9 cells, it is obviously less than that of Vip3Aa protoxin (S7B Fig). So we think that the Vip3Aa protoxin plays a major toxic role in cytotoxicity assays. Furthermore, we found that endocytosis of Vip3A was almost completely inhibited after treated with dynasore (Fig 5B). Meanwhile, the toxicity of Vip3Aa was decreased clearly but not reduced accordingly (Fig 7A). It implied that endocytosis is critical for Vip3Aa to exert its toxic effects, but it may not be responsible for all the toxicity of Vip3Aa. Recently, Tabashnik et al. proposed a new model for Bt mode of action named “dual model”, where both the protoxin and activated Cry toxin forms can kill insects, with each form exerting its toxic effect via a different pathway [49]. This contrasts with what “classical model” in which protoxins are inactive. Whether Vip3Aa protoxin and the activated toxin use the different mechanism of action and whether Vip3A have other mechanisms of insecticide need further study.

To date, SR-C has only been described in *Drosophila*. The present study cloned and identified another *SR-C* gene in *S*. *frugiperda*. Moreover, we also cloned two other fragments from *S*. *exigua* cells, which have high sequence and structural homology with Sf-SR-C. This indicated that SR-C also exists in *S*. *exigua* and further extends the range of SR-C in insects. Our results showed that SR-C can be detected in Sf9 cells and *S*. *exigua* cells, as well as in *Drosophila* cells. However, only the former two types of cells have high affinity for Vip3Aa (Fig 1A and S3A Fig). We hypothesized that subtle differences in the sequence and the three-dimensional structure of the protein might influence their interaction with Vip3Aa. Consistent with our conjecture, Vip3Aa-Flag can bind to Sf-CCP but not to DM-CCP (Fig 4D and 4E). Furthermore, from the sequence alignments, we found that Sf-SR-C has no sequence and structural homology with known proteins from vertebrates, as well as with known Cry toxins receptors. The results presented here provide a plausible molecular basis for the lack of toxicity of Vip3A toxins toward non-target insects and vertebrates, and strongly support its use as a safe biopesticide. In addition, because SRs play a crucial role in innate immunity and in the pathogenesis of various diseases in mammals [34], our study might extend our understanding of SR-C proteins and provide other avenues for studying host-pathogen interactions.

What’s more, although reducing the expression of the *Se-SR-C* gene clearly reduced the toxicity of Vip3Aa to the larvae compared with that of the control (Fig 3C), the effect sizes between the larvae of *Se-SR-C* gene silencing and the control groups were not as obvious as expected. This implied that there may be other receptors for Vip3Aa contributing to the overall toxicity. Just like several receptors for Cry toxin have been discovered [1, 7, 8], some reports also found Vip3Aa could bind to different molecular weight proteins in the brush border membrane vesicles of susceptible insects, such as the 55, 65, 80, 100 and 110 kDa proteins [15, 50–52], which further indicated the existence of different kinds of receptors for Vip3Aa. Moreover, we have identified 36 other proteins besides the ribosomal proteins and Sf-SR-C from the extracted Sf9 cell membrane proteins which could interact with the Vip3Aa. Whether or not there are other receptors play roles, sequentially or simultaneously, in killing insect process, further in-depth studies are needed.

In conclusion, the present study identified and confirmed Sf-SR-C as the receptor for Vip3Aa, proved the CCP and MAM domains of Sf-SR-C interact with Vip3Aa, analyzed the binding specificity between Vip3Aa and Sf-SR-C, and certified Sf-SR-C mediate the internalization of Vip3Aa via endocytosis. Our results contribute to the understanding of the mode of action Vip3Aa and significantly facilitate the further study of its insecticidal mechanism and application.

## Materials and methods

### Bacterial strains, cell lines and insects

*E*. *coli* DH5α for plasmid constructions and *E*. *coli* BL21 (DE3) for protein purification were cultured at 37 °C in lysogeny broth (LB) or agar. Bt9816C was previously isolated and maintained in our laboratory for generation of Vip3Aa [53]. The *Drosophila* S2 cells, *S*. *frugiperda* Sf9 cells and *S*. *exigua* cells (IOZCAS-Spex-II-A) were maintained and propagated in Sf-900 II SFM (Invitrogen) or SFX-Insect (HyClone) culture medium at 27 °C. *Spodoptera exigua* and *Drosophila* strains were used for the bioassays.

### Drosophila genetics

*Drosophila* genotypes used were: ***esg***^***ts***^: *esg*-*Gal4*, *tub*-*Gal80*^*ts*^, *UAS*-*GFP/cyo*; *Tm2*/*Tm6B*. ***UAS*-*SR*-*C***^***vk33***^: SP/Cyo; *10*×*UAS*-*SR*-*C*^*vk33*^/*Tm6B*. ***esg***^***ts***^**>*SR*-*C***^***vk33***^: *esg*-*Gal4*, *tub*-*Gal80*^*ts*^, *UAS-GFP*/*Cyo*; *UAS-SR*-*C*^*vk33*^/*Tm2*.

To ectopically express Sf-SR-C in *Drosophila*, the primers pJF-Sf-SR-C-F and pJF-Sf-SR-C-R were used to clone the *Sf-SR-C* gene, which was then recombined with the linearized vector pJFRC2-10XUAS-IVS-mCD8::GFP (Addgene plasmid #26214) using a ClonExpress II One Step Cloning Kit (Vazyme). Transgenic lines were established through microinjection of the transgene DNA into embryos of PhiC31-mediated chromosome-integrated *Drosophila* strains PBac {y[+]-attP-3B} VK00033 [54].

### Reagents and antibodies

Primary antibodies: Mouse anti-Flag (Cell Signaling 8146), rabbit anti-V5 (Cell Signaling 13202), rabbit anti-His (Cell Signaling 12698), anti-Sf-SR-C-N polyclonal antibodies were generated by immunizing rabbits with purified GST-SR-C-N. Secondary antibodies: goat anti-mouse IgG-HRP conjugate (Santa Cruz sc-2005), goat anti-rabbit IgG-HRP conjugate (Cell Signaling 7074), rabbit anti-GST (Polyclonal, Bioss bs-2735R). The primary antibodies and secondary antibodies were used at 1: 1000 for western blotting. For immunostaining assays, Anti-V5-Dylight 488 conjugate (Invitrogen MA5-15253-D488, 1:200) and Alexa Fluor 488 goat anti-rabbit IgG (Cell Signaling 4412, 1:200) were used.

Inhibitors: Dynasore (dynamin inhibitor, TargetMol T1848, 7.5μM), chlorpromazine (clathrin-mediated endocytosis inhibitor, Millipore 215921, 40μM), monodansylcadaverine (clathrin-mediated endocytosis inhibitor, Sigma D4008, 150μM), nystatin (sequesters cholesterol, Millipore 475914, 20μM), cholesterol-oxidase (oxidize cholesterol, Millipore 228230, 4unit/ml), amiloride (macropinocytosis inhibitor, Millipore 129876, 150μM), Cytochalasin D (macropinocytosis inhibitor, Millipore 250225, 500nM), LY294002 (broad PI(3)K inhibitor, Cell Signaling 9901s, 50μM), and wortmannin (broad PI(3)K inhibitor, Cell Signaling 9951s, 2μM). Cells were treated with the inhibitors for 1 h at 27°C before Vip3Aa-RFP was added.

### Protein expression and purification

For the expression of Vip3Aa protoxin, the *vip3Aa* gene was cloned in pET-28a(+) vector (Novagen) using oligonucleotide primer Vip-F and Vip-R (plasmid pET-Vip), resulting in a His_6_ fusion. For the expression of Vip3Aa-RFP, the *vip3Aa* gene and *rfp* gene were amplified using oligonucleotide primer pairs Vip-RFP-Up-F and Vip-RFP-Up-R, and Vip-RFP-Do-F and Vip-RFP-Do-R, respectively. Then the two gene fragments were ligated into the pET-28a (+) vector (Novagen) using a pEASY-Uni Seamless Cloning and Assembly Kit (TransGen) after digesting the vector with *Nco*I and *Xho*I (plasmid pET-Vip-RFP), resulting in a His_6_ fusion. The plasmids used to express Vip3Aa-Flag (pET-Vip-flag) was constructed similarly to plasmid pET-Vip-RFP, using oligonucleotide primer Vip-flag-F and Vip-flag-R. The plasmids used to express RFP (pET-RFP), Sf-CCP (pET-Sf-CCP) and Dm-CCP (pET- Dm-CCP) were constructed similarly to plasmid pET-Vip, using oligonucleotide primer pairs RFP-F and RFP-R, Sf-CCP-F and Sf-CCP-R, and Dm-CCP-F and Dm-CCP-R, respectively.

For the expressing of Sf-SR-C-N fused with glutathione-S-transferase (GST), the *Sf-SR-C-N* gene was amplified using primer SR-C-N-F and SR-C-N-R. The amplification product was inserted into the pGEX-6P-1 (GE Healthcare) vector using a pEASY-Uni Seamless Cloning and Assembly Kit (TransGen) after digesting the vector with *Bam*HI and *Xho*I (plasmid pGEX-SR-C-N). The plasmids used to express SR-F-1 (pGEX-SR-F-1), SR-F-2 (pGEX-SR-F-2), and SR-F-3 (pGEX-SR-F-3) were constructed similarly to plasmid pGEX-SR-C-N, using oligonucleotide primer pairs SR-F-1-F and SR-F-1-R, SR-F-2-F and SR-F-2-R, and SR-F-3-F and SR-F-3-R, respectively.

Plasmids were transformed into *E*. *coli* BL21 (DE3) (Invitrogen) for expression and purification [55]. His-tagged proteins was purified by using cOmplete His-Tag Purification Resin (Roche), whereas GST- tagged proteins was purified by using GST-Sepharose affinity column (GE Healthcare). The purified protein was dialyzed in buffer containing 25 mM Tris-Hcl (pH 8.0), 150 mM NaCl and 5% glycerol at 4 °C with three buffer changes. The purified Vip3Aa is used for cytotoxicity assays.

All the primers and plasmids used in this study are shown in S1 and S2 Tables.

### Microscale thermophoresis (MST) assay

MST was used to determine the binding affinity between Vip3Aa protoxin and Sf-SR-C protein fragments. Briefly, purified proteins were dialyzed into 25 mM Hepes (pH 7.5), 150 mM NaCl, and 0.05 (v/v) % Tween-20. The purified Vip3Aa was labeled with the Monolith NT Protein Labeling Kit (Cat # L008) according to the supplied labeling protocol. 10 nM labeled Vip3Aa were incubated with 0.3 nM to 10 μM Sf-SR-C protein fragments for 20 min at RT respectively. Samples were then loaded into standard treated capillaries and analyzed with a NanoTemper Monolith NT.115 Pico (NanoTemper Technologies GmbH, Munich, Germany) at 25°C. Furthermore, the laser power was set to 10% and the LED power was set to 60%. Normalization of the fluorescence signal and fitting to the Hill equation were performed using the software MO Affinity Analysis v2.2.2 (NanoTemper). For each sample, the whole procedure was performed three times to yield independent triplicates.

### RNA extraction, cDNA cloning, and real-time PCR

Total RNA was isolated from cultured cells or *S*. *exigua* midgut using RNAiso Plus (Takara). cDNA was synthesized using a Transcriptor High Fidelity cDNA Synthesis Kit (Roche). Quantification of the cDNA was carried out using SYBR Premix Ex Taq II (Takara) and analyzed by using StepOne software (Applied Biosystems) as previously described [55]. The actin gene acted as the endogenous control. The relative amount of cDNA was calculated according to the 2^−ΔΔ*CT*^ method [56]. Data were analyzed from three independent experiments and are shown as means ± SD.

### Plasmid construction, transfection, and dsRNA preparation

Plasmids used for *Sf-SR-C* gene silencing experiments were constructed as described by Katsuma et al. [25]. Fragments of the *Sf-SR-C* gene (nucleotides [nt] 294 to 803, dsRNA1s) and 400 bp from the complementary strand of the *Sf-SR-C* gene (nt 693 to 294, dsRNA1as) were amplified by using the primer sets SRi1-Up-F and SRi1-Up-R (for dsRNA1s) or SRi1-Do-F and SRi1-Do-R (for dsRNA1as). dsRNA1s was designed to be 110 bp longer than the dsRNA1as. dsRNA1s and dsRNA1as were inserted in tandem into the pIZT/V5-His vector using a pEASY-Uni Seamless Cloning and Assembly Kit (TransGen) after digesting the vector with KpnI and AgeI (pIZT-SRi1). In the same way, we constructed pIZT-SRi2 using the primer sets SRi2-Up-F and SRi2-Up-R for dsRNA2s (nt 1081–1590) or SRi2-Do-F and SRi2-Do-R for dsRNA2as (nt 1480–1081). We generated stable *Sf-SR-C* gene silencing Sf9 cells lines by transfection with pIZT-SRi1 or pIZT-SRi2 using the Cellfectin II reagent (Invitrogen) and PLUS Reagent (Invitrogen). At 2 d after transfection, zeocin (500 μg/mL) was added into the medium. Two to three weeks after drug selection, we examined the expression level of the *Sf-SR-C* gene by qRT-PCR analysis by using the primers SR-RT-F and SR-RT-R. The vectors pIZT-SR-C, pAc-SR-C, and pAc-Sf-S2, which were used to express the Sf-SR-C or Sf-S2, were transfected into Sf9 cells or S2 cells to express the targeted proteins using Cellfectin II reagent and PLUS Reagent.

The plasmid pET-Se-SRi and pET-Hypi were constructed as the pIZT-SRi1 by using the primer sets pET-SRi-Up-F and pET-SRi-Up-R for dsRNA3s (nt 1–870), pET-SRi-Do-F and pET-SRi-Do-R for dsRNA3as (nt 718–1), Hypi-Up-F and Hypi-Up-R for dsRNA4s (620bp), and Hypi-Do-F and Hypi-Dp-R for dsRNA4as (500bp). Then the dsRNA3s and dsRNA3as or dsRNA4s and dsRNA4as were inserted in into the pET28a vector. The pET-Se-SRi and pET-Hypi were transformed into the HT115 (DE3) strain, which lacks RNase III activity for dsRNA expression, as described by Tian et al. [26].

### Mass spectrometry

The purified Vip3Aa protoxin was labeled with biotin using an EZ-Link Sulfo-NHS-SS-Biotinylation Kit. (Thermo Scientific). The membrane proteins of Sf9 cells were extracted using a ProteoExtract Transmembrane Protein Extraction Kit (Novagen). Streptavidin Mag Sepharose beads (50 μL) (GE Healthcare) were washed and incubated with 0.2 mg biotin labeled Vip3Aa (Bio-Vip3Aa) for 1 h at 4 °C and washed three times with TBS to remove unbound proteins. The Vip3Aa tagged beads were then incubated with 1 mL of extracted Sf9 cell membrane proteins for 3 h at 4 °C and washed five times with washing buffer (TBS + 2 M urea). The precipitants were boiled with SDS loading buffer and subjected to SDS-PAGE. After cutting out the band representing Vip3Aa, the remaining bands were sent for LC-MS/MS (tandem mass spectroscopy) analysis.

### Western blotting and immunoprecipitation

The targeted sample was resolved by SDS-PAGE and transferred onto a Polyvinylidene fluoride (PVDF) membrane (Millipore). Primary antibody and HRP-coupled secondary antibody were used to detect the sample. The membrane was visualized using Immobilon Western chemiluminescent HRP Substrate (Millipore).

Cells were collected and lysed in 0.5 ml radio immunoprecipitation assay buffer (Cell Signaling 9806S) for 30 min on a rotor at 4 °C. After centrifugation at 12 000× *g* for 15 min, the lysate (30μL) was co-incubated with Vip3Aa-Flag (10 μg) for 2 h at 4 °C. The sample was immunoprecipitated with 5 μL anti-V5 antibody overnight at 4 °C, and 40 μL of protein G agarose beads (Santa Cruz) were washed and then added for additional 4 h. Thereafter, the precipitants were washed five times with washing buffer (3.2 mM Na_2_HPO_4_, 0.5 mM KH_2_PO_4_, 1.3 mM KCl, 135 mM NaCl, pH 7.4), and the immune complexes were boiled with loading buffer for 6 min and then analyzed by western blotting.

### Dot blotting, ligand blotting and pull-down

Five microliter of different regions of the Sf-SR-C protein (0.1 nmol) were dotted onto a PVDF membrane. After blocking with 5% skimmed milk in phosphate buffer solution with 0.05% tween-20(PBST), the membrane was incubated in Vip3Aa-flag (100 nM) for 1 h at room temperature, and washed at least three times using PBST. Vip3Aa without Flag-tag (500-fold excess) was used in the competition assays. The following steps are consistent with western blotting.

Ten microliter of Sf9-pIZT-SR-C cells lysate were subjected to SDS-PAGE and then transferred to PVDF membrane. After blocking with 5% skimmed milk in PBST, the membrane were incubated in Vip3Aa-flag (100 nM) for 2 h at room temperature, and washed at least three times using PBST. Vip3Aa without Flag-tag (200-fold excess) was used in the competition assays. The following steps are consistent with western blotting.

Different parts of the Sf-SR-C protein fused with (GST) (0.4 nmol) were incubated with GST-Sepharose affinity beads at 4 °C for 3 h and then washed three times with PBS to remove unbound proteins. Beads were incubated with Vip3Aa-flag (100 nM) and washed five times with PBS. The precipitated components were boiled with sample buffer for 10 min and analyzed by western blotting.

### Cytotoxicity assays

Cell viability assays were performed using the CCK-8 Counting Kit (Dojindo). Briefly, cells with a density of 5 × 10^4^ cells per ml were seeded into 96-well culture plates separately. After overnight incubation, the cells were treated with Vip3Aa protoxin (50 μg/mL) for 48 h. WST-8 reagent was then added to each well. After incubating at 27 °C for 2 h, the absorbance was measured in microplate reader (PerkinElmer) at 450 nm. Treatment with sterile dialysis buffer was used as a control. All tests were performed in triplicate and were repeated at least three times. Cell viability (%) = average absorbance of treated group / average absorbance of control group × 100%.

### Immunostaining and confocal microscopy

Cells were grown to 60–80% confluence in Laser confocal culture dishes. After treatment, cells were washed three times with PBS to remove unbound ligands, and fixed with freshly prepared 4% paraformaldehyde at 37 °C for 30 min. For co-localization experiments, cells were then permeabilized (0.2% Triton X-100) and immunostained (primary and secondary antibodies were diluted in 5% skimmed milk powder). Cellular cortical actin and nuclei were labeled for 30 min with fluorescein isothiocyanate (FITC)-phalloidin (Sigma) and DAPI (Sigma) respectively. Cell images were captured using a Zeiss.LSM710 confocal microscope.

### Bioassay

*S*. *exigua*:

- A randomized block design was used for this bioassay experiment. An artificial diet was prepared and put into the wells of a 24-well cell culture plate (about 5 mm thick per hole).
- The bacterially expressed dsRNAs were prepared as described by Tian et al. [26] using the strain HT-pET-Se-SRi. The strains HT-pET-Hypi and HT-pET28a as control. Each hole was overlaid with 20 μL bacterial suspension and put it in the room temperature for about 1h to let the diet dry. (For the first three days, the number of bacteria on the diet were 2 × 10^7^ per well. For the next four days, the number of bacteria on the diet were 4 × 10^7^ per well.)
- Select the 1^st^ instar larvae, put them on the diet coated with bacteria and continue feeding for at least seven days. For the first three days, six larvae were reared in each hole. For the next four days, three larvae were reared in each hole. All diets were replaced daily.
- After continuous feeding the *S*. *exigua* larvae with bacterially expressed dsRNA for seven days, about twenty 3^st^ instar larvae were selected (about 5–8 mm) from each group, and the transcription level of the *Se-SR-C* gene in the midgut of the larvae was detected by qRT-PCR.
- Twenty 3^st^ larvae of similar sizes were then selected and put onto the diet overlaid with Vip3Aa (0.8 μg/cm^2^) and the bacteria cells (4 × 10^7^ per well). On average, one larvae was placed in each well and were cultured at 28 °C for 5 days. All diets were replaced every two days.
- The larvae are considered dead when they do not move in the case of a slightly shaken culture plate. Count the number of deaths every day and repeat the experiments above 3 times.

*Drosophila*:

- The adult flies of different strains (*esg*^*ts*^, *UAS-SR-C*^*vk33*^ *and esg*^*ts*^*>SR-C*^*vk33*^) were transferred to fresh medium respectively (At least ten tubes of *Drosophila* were prepared for each strain, and at least 20 flies per tube) and reared at 25 °C for 4 days. Then the strains of *esg*^*ts*^ (a) and *UAS-SR-C*^*vk33*^ (b) were also transferred to 29 °C. The flies of *esg*^*ts*^*>SR-C*^*vk33*^ were divided into two groups on average, one group was cultured at 29 °C to inactivate Gal80^ts^, allowing Gal4 to activate *UAS* transgenes (c), the other group was shifted to 18 °C to restrict Gal4 activity (d). Continue to culture for at least three days.
- The adult flies of each strain were transferred to the new culture tube respectively, retaining the remaining larvae. 2^st^ instar larvae were selected from the remaining larvae of each strain separately and put into 48-well cell culture plates which filled with 200 μL sterile PBS in each well (Five larvae were placed in each well on average and each group included at least 20 larvae). The larvae in each sample were prepared in two groups (a1 and a2, b1 and b2, c1 and c2, d1 and d2).
- Then Vip3Aa (20 μg) was added into every well of one group (a1, b1, c1 and d1) and equivoluminal dialysis buffer was added into another group as the parallel control group (a2, b2, c2 and d2). These larvae were culture at 29 °C (a1 and a2, b1 and b2, c1 and c2) or 18 °C (d1 and d2) for 48 h.
- The larvae are considered dead when they did not move in the case of a slightly shaken culture plate. The number of deaths per 12 h were counted and the experiment was repeated three times

### Statistical analysis

Experiments were performed at least three times independently. All statistical data were calculated with SPSS software. (v.22.0). For comparisons of the means of two groups, two-tailed t test was used. For comparisons of multiple groups with a control group, one-way ANOVA method was used. Significance of mean comparison is annotated as follow: ns, not significant; *P<0.05; **P<0.01; ***P<0.001.

## Supporting information

S1 Fig Analysis of Vip3Aa-RFP toxicity to Sf9 cells, SDS-PAGE analysis of Vip3A protoxins, identification of Sf-SR-C peptides that bind to Vip3A and affinity magnetic bead method to detect the binding of Vip3Aa and Sf-SR-C. (A) Microscopic views of Sf9 cells treated with RFP (control) and Vip3Aa-RFP (25μg/ml) for 48 h, respectively. (B) The purified Vip3Aa-His (Vip3Aa) and Vip3Aa-Flag-His (Vip3Aa-Flag), as well as biotin labeled Vip3Aa-His (Bio-Vip3Aa) were separated by SDS-PAGE. (C) Identified mass spectrometry spectrums of Sf-SR-C peptides: CDLYAEATGYR, YTLVGNK, and LLSPVYDAELAK. (D) Bio-Vip3Aa-Flag or Bio-ChiB-Flag was incubated with Sf9-pIZT-SR-C cells lysate, immunoprecipitated with Streptavidin Mag Sepharose, and Sf-SR-C in the elution was detected by immunoblotting with anti-V5 antibody.
(TIF)

S2 Fig Schematic diagram of the recombinant plasmids for dsRNA expression. (A) Schematic diagram of pIZT-SRi1. (B) Schematic diagram of pIZT-SRi2. (C) Schematic diagram of pET-Se-SRi.
(TIF)

S3 Fig Sf-SR-C acts as the receptor of Vip3Aa *ex vivo*. (A) Confocal microscopy images of S2 cells and IOZCAS-Spex-II-A cells treated with Vip3Aa-RFP (red fluorescent protein) (4.5μg/mL) for 6 h. Cells were counterstained with DAPI (2-(4-amidinophenyl)-1H-indole-6-carboxamidine; blue) and FITC (fluorescein isothiocyanate)-phalloidin (green). Scale bar, 10 μm. (B) Western blotting showing the expression of Sf-SR-C and Sf-S2 in S2 cells using an anti-V5 antibody. (C) S2 cells were transfected with Sf-SR-C. 48 h after transfection, cells were exposed to Vip3Aa-RFP or RFP (red), fixed, and then immunostained with Dylight 488-conjugated anti-V5 antibodies (green). Scale bar, 10 μm.
(TIF)

S4 Fig Sf-SR-C as the receptor for Vip3Aa. (A) Total RNA was extracted from bacteria HT115 strains containing the plasmid pET-Se-SRi, pET-Hypi and the blank plasmid pET28a after IPTG induction to show the dsRNA. The position of dsRNA produced is indicated with an arrowhead. (B) The survival rate of 2^st^ instar *S*. *exigua* larvae on the diet containing 4 × 10^7^ bacteria (the strain HT-pET-Se-SR) per well for 6 days, the larvae on the diet without bacteria as control. The survival rates of each group were analyzed every day. Data were showed as mean ± SD (n = 20). (C) The survival rate of the *Drosophila* larvae (*esg*^*ts*^) in different concentrations of Vip3Aa toxin (10, 20, 50, and 100 μg/ml). The survival rates of each group were analyzed every 12 h. Data were showed as mean ± SD (n = 20). (D) The survival rate of the *Drosophila* larvae (*esg*^*ts*^*>SR-C*^*vk33*^) in PBS for 3 days (n = 20), and the survival rates were analyzed every 12 h. Data were showed as mean ± SD.
(TIF)

S5 Fig Quantifying the amount of Vip3Aa-RFP on Sf9 cells membrane. Sf9 cells were pre-treated with or without dynasore for 1 h and then co-incubated with Vip3Aa-RFP (10 μg/ml) for 6 h. The amount of Vip3Aa-RFP on Sf9 cell membrane were assessed and quantified in a blind fashion by ImageJ (n = 60 cells per sample). Data are expressed as the mean ± SD from three experiments; * P < 0.05 by two-tailed Student’s t tests.
(TIF)

S6 Fig Western blotting to show the Vip3Aa protein by anti-Vip3Aa polyclonal antibody. Lane 1, the Vip3Aa after incubating with Sf9 cells for 12 h. Lane 2, the Vip3Aa in the medium for 12 as control.
(TIF)

S7 Fig The activated Vip3Aa against Sf9 cells. (A) The purified Vip3Aa protoxin and activated Vip3Aa protein (Vip3Aa-199) were separated by SDS-PAGE. (B) Cell viability of Sf9 cells separately treated with Vip3Aa protoxin (50 μg/mL) and activated Vip3Aa protein (Vip3Aa-199) (50 μg/mL) for 60 h. Data are expressed as the mean ± SD from three independent experiments; *** P < 0.001 by two-tailed Student’s t tests.
(TIF)

S1 Table Primers used in this study. (PDF)

S2 Table Plasmids used in this study. (PDF)

S1 Dataset The identified proteins from the protein sequence database according to the MS/MS spectra. (XLS)
