# Supplementary figures and images for: Scavenger receptor-C acts as a receptor for Bacillus thuringiensis vegetative insecticidal protein Vip3Aa and mediates the internalization of Vip3Aa via endocytosis
Source: PLoS Pathog. 2018 Oct 4;14(10):e1007347. doi: 10.1371/journal.ppat.1007347 (PMC6191154; doi:10.1371/journal.ppat.1007347)

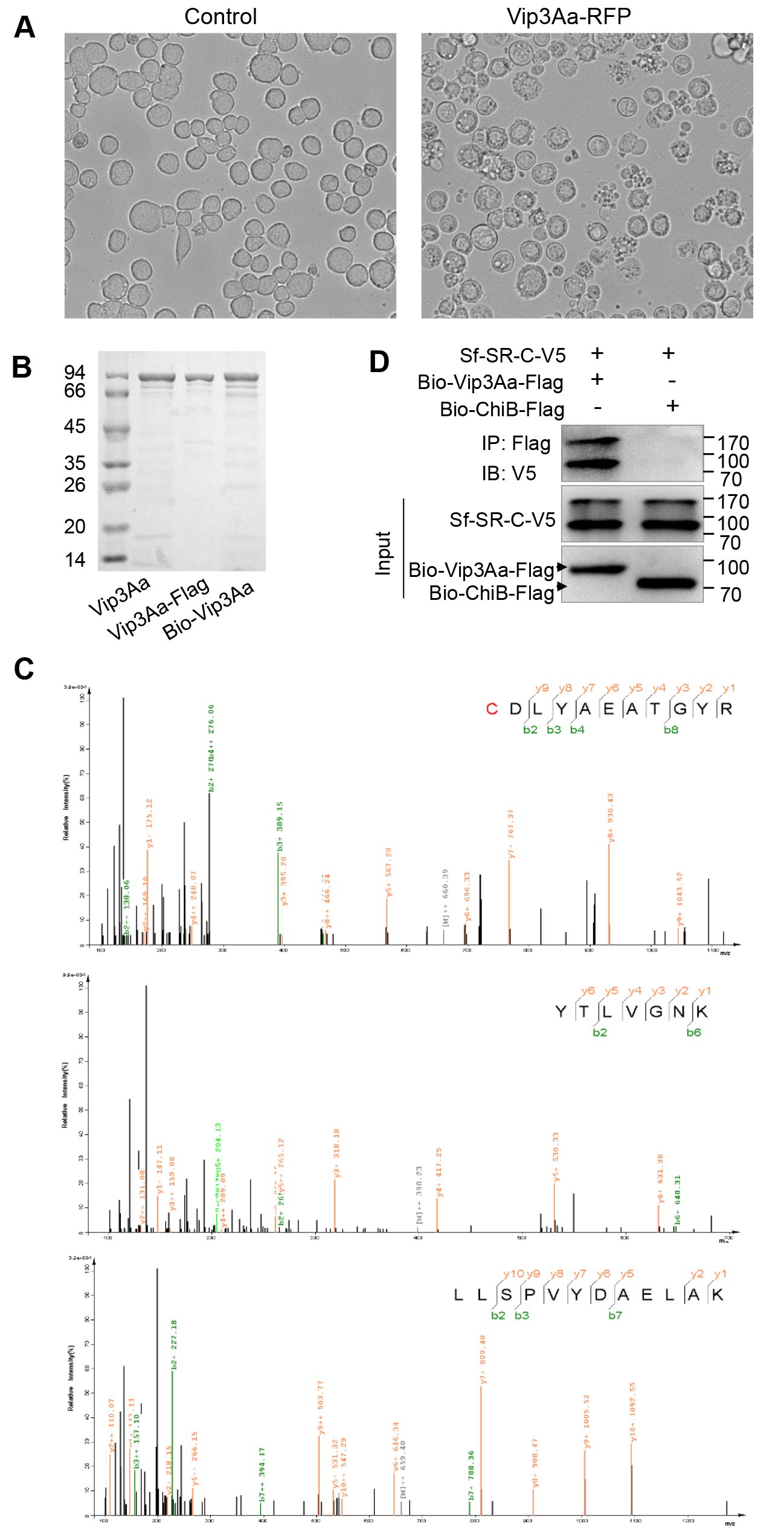

Supplement: S1 Fig — (A) Microscopic views of Sf9 cells treated with RFP (control) and Vip3Aa-RFP (25μg/ml) for 48 h, respectively. (B) The purified Vip3Aa-His (Vip3Aa) and Vip3Aa-Flag-His (Vip3Aa-Flag), as well as biotin labeled Vip3Aa-His (Bio-Vip3Aa) were separated by SDS-PAGE. (C) Identified mass spectrometry spectrums of Sf-SR-C peptides: CDLYAEATGYR, YTLVGNK, and LLSPVYDAELAK. (D) Bio-Vip3Aa-Flag or Bio-ChiB-Flag was incubated with Sf9-pIZT-SR-C cells lysate, immunoprecipitated with Streptavidin Mag Sepharose, and Sf-SR-C in the elution was detected by immunoblotting with anti-V5 antibody. (TIF) [file ppat.1007347.s001.tif]

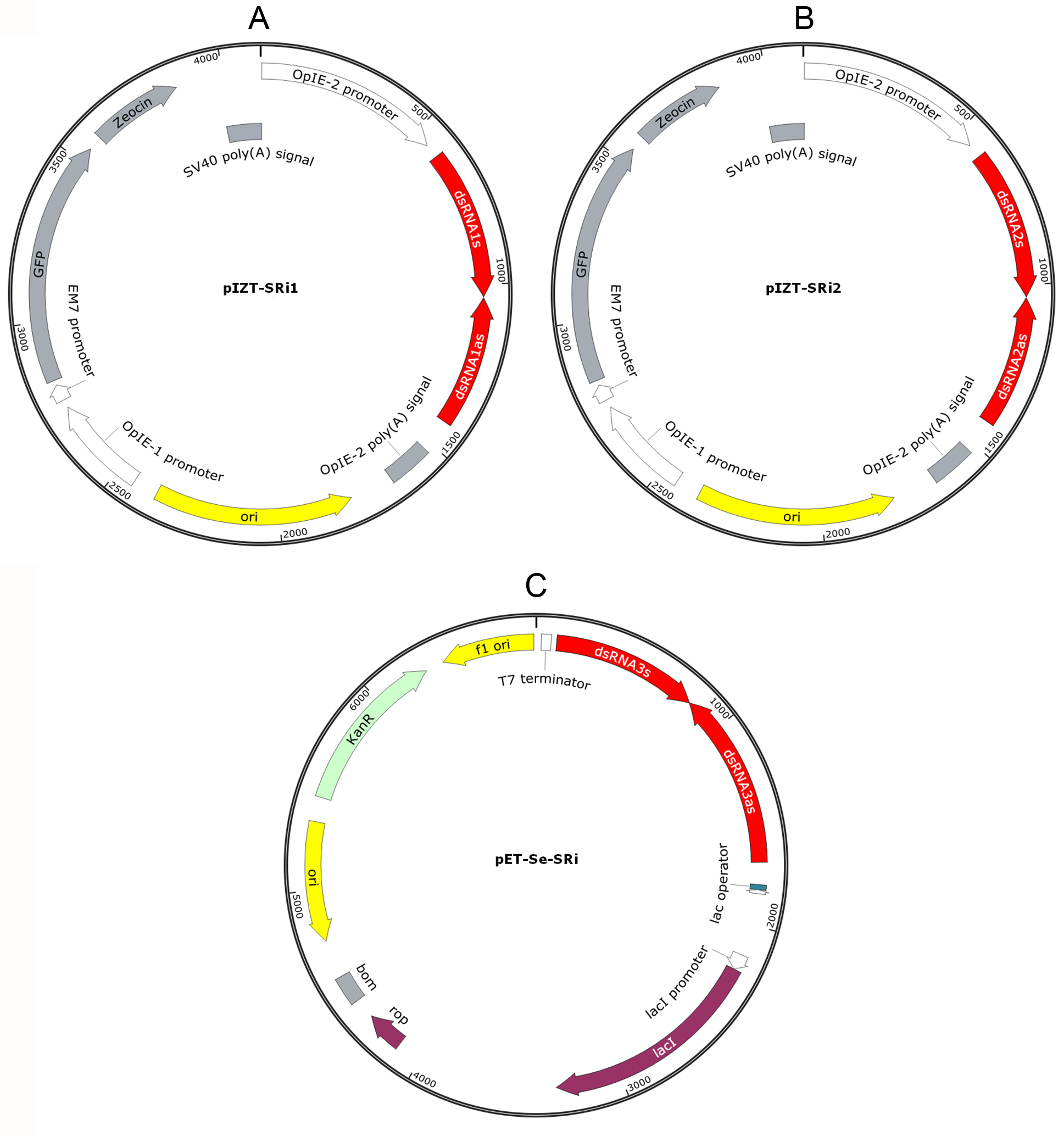

Supplement: S2 Fig — (A) Schematic diagram of pIZT-SRi1. (B) Schematic diagram of pIZT-SRi2. (C) Schematic diagram of pET-Se-SRi. (TIF) [file ppat.1007347.s002.tif]

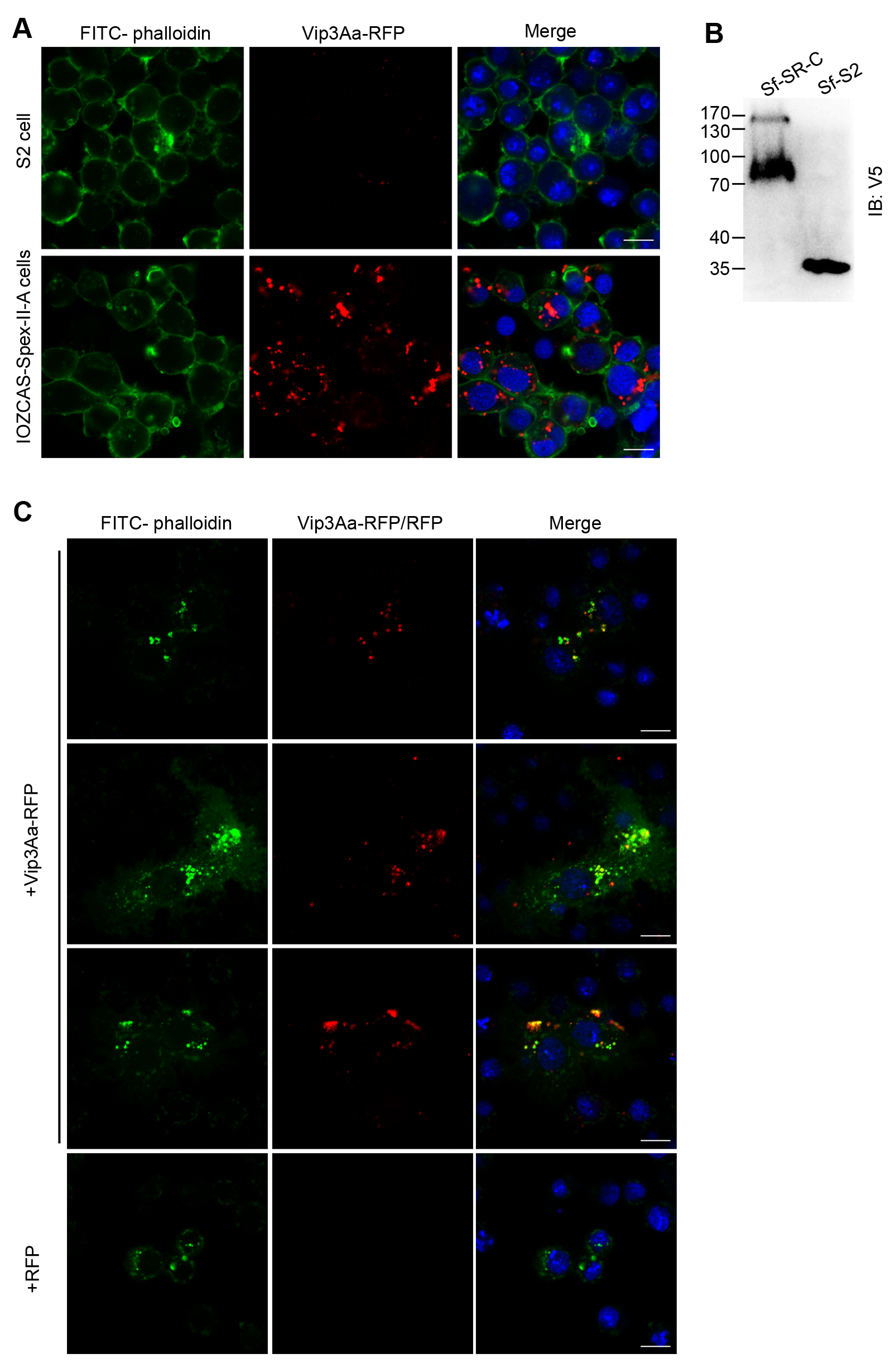

Supplement: S3 Fig — (A) Confocal microscopy images of S2 cells and IOZCAS-Spex-II-A cells treated with Vip3Aa-RFP (red fluorescent protein) (4.5μg/mL) for 6 h. Cells were counterstained with DAPI (2-(4-amidinophenyl)-1H-indole-6-carboxamidine; blue) and FITC (fluorescein isothiocyanate)-phalloidin (green). Scale bar, 10 μm. (B) Western blotting showing the expression of Sf-SR-C and Sf-S2 in S2 cells using an anti-V5 antibody. (C) S2 cells were transfected with Sf-SR-C. 48 h after transfection, cells were exposed to Vip3Aa-RFP or RFP (red), fixed, and then immunostained with Dylight 488-conjugated anti-V5 antibodies (green). Scale bar, 10 μm. (TIF) [file ppat.1007347.s003.tif]

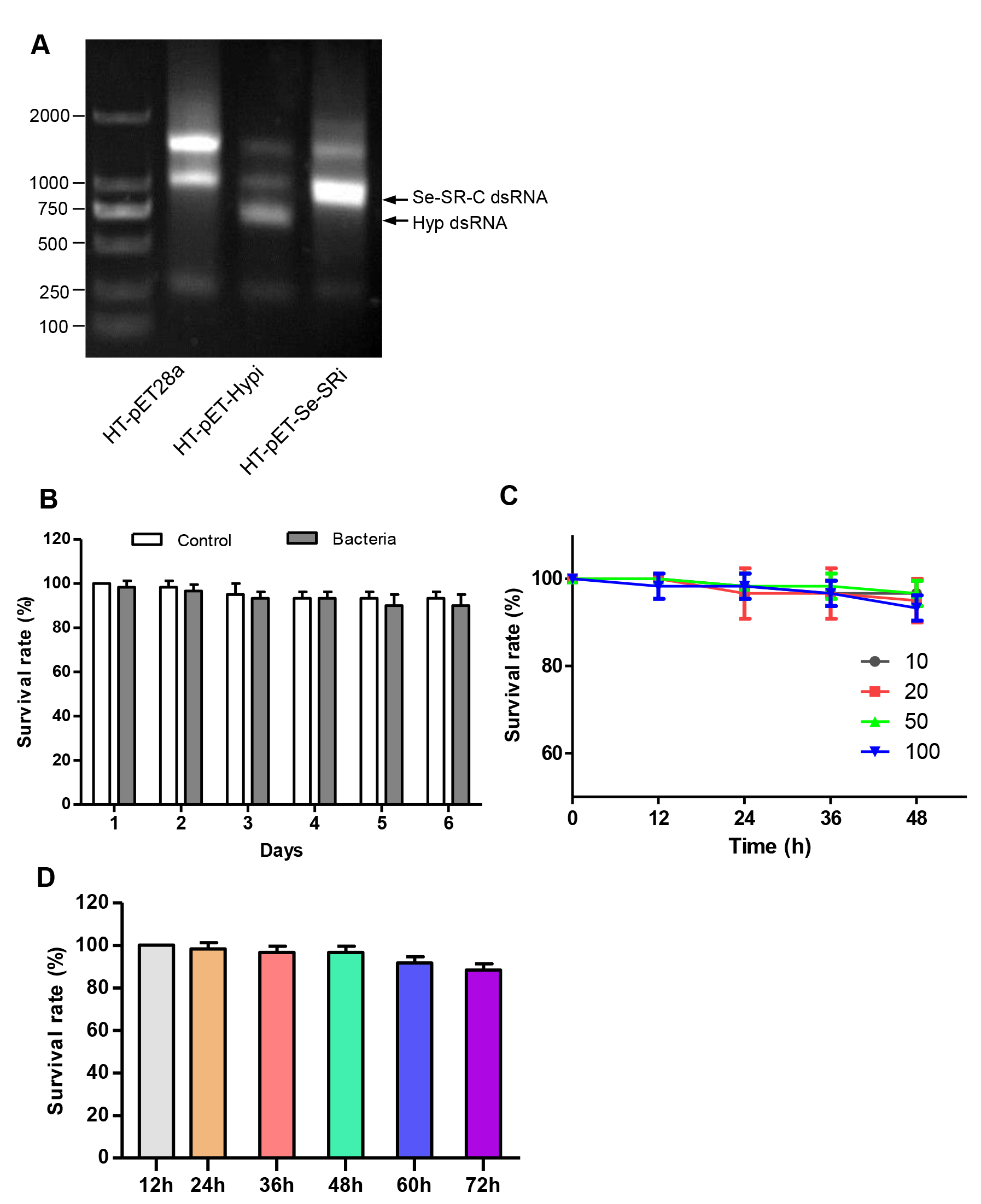

Supplement: S4 Fig — (A) Total RNA was extracted from bacteria HT115 strains containing the plasmid pET-Se-SRi, pET-Hypi and the blank plasmid pET28a after IPTG induction to show the dsRNA. The position of dsRNA produced is indicated with an arrowhead. (B) The survival rate of 2st instar S. exigua larvae on the diet containing 4 × 10^7^ bacteria (the strain HT-pET-Se-SR) per well for 6 days, the larvae on the diet without bacteria as control. The survival rates of each group were analyzed every day. Data were showed as mean ± SD (n = 20). (C) The survival rate of the Drosophila larvae (esgts) in different concentrations of Vip3Aa toxin (10, 20, 50, and 100 μg/ml). The survival rates of each group were analyzed every 12 h. Data were showed as mean ± SD (n = 20). (D) The survival rate of the Drosophila larvae (esgts>SR-Cvk33) in PBS for 3 days (n = 20), and the survival rates were analyzed every 12 h. Data were showed as mean ± SD. (TIF) [file ppat.1007347.s004.tif]

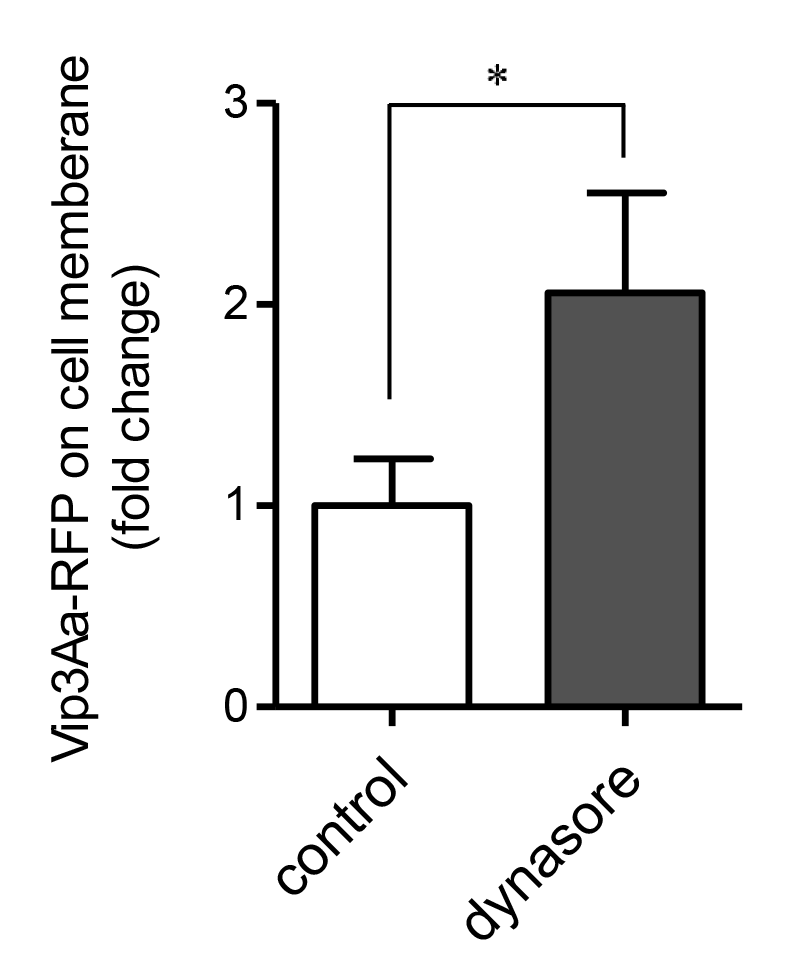

Supplement: S5 Fig — Sf9 cells were pre-treated with or without dynasore for 1 h and then co-incubated with Vip3Aa-RFP (10 μg/ml) for 6 h. The amount of Vip3Aa-RFP on Sf9 cell membrane were assessed and quantified in a blind fashion by ImageJ (n = 60 cells per sample). Data are expressed as the mean ± SD from three experiments; * P < 0.05 by two-tailed Student’s t tests. (TIF) [file ppat.1007347.s005.tif]

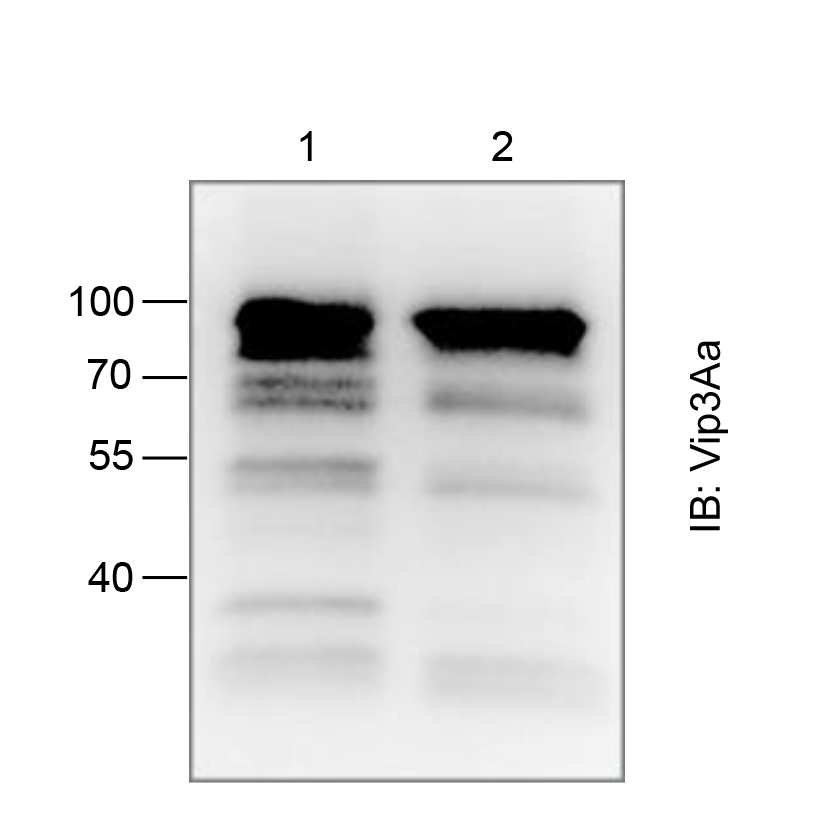

Supplement: S6 Fig — Lane 1, the Vip3Aa after incubating with Sf9 cells for 12 h. Lane 2, the Vip3Aa in the medium for 12 as control. (TIF) [file ppat.1007347.s006.tif]

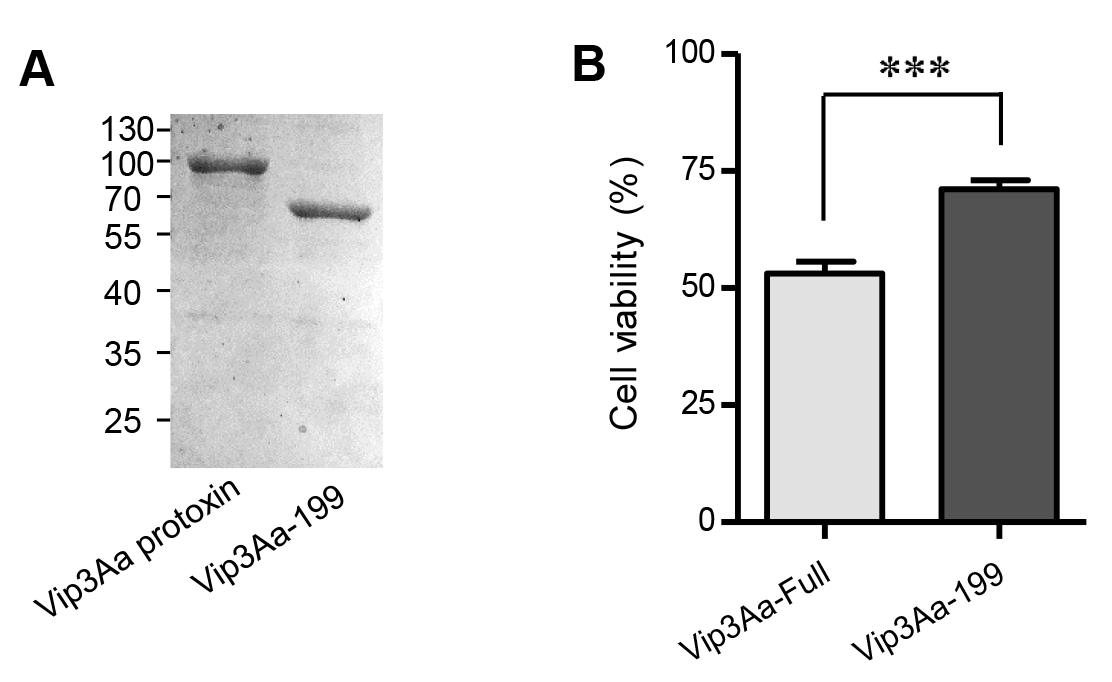

Supplement: S7 Fig — (A) The purified Vip3Aa protoxin and activated Vip3Aa protein (Vip3Aa-199) were separated by SDS-PAGE. (B) Cell viability of Sf9 cells separately treated with Vip3Aa protoxin (50 μg/mL) and activated Vip3Aa protein (Vip3Aa-199) (50 μg/mL) for 60 h. Data are expressed as the mean ± SD from three independent experiments; *** P < 0.001 by two-tailed Student’s t tests. (TIF) [file ppat.1007347.s007.tif]
