# Supplementary material for: Scavenger receptor-C acts as a receptor for Bacillus thuringiensis vegetative insecticidal protein Vip3Aa and mediates the internalization of Vip3Aa via endocytosis
Source: PLoS Pathog. 2018 Oct 4;14(10):e1007347. doi: 10.1371/journal.ppat.1007347 (PMC6191154; doi:10.1371/journal.ppat.1007347)
Supplement: S1 Table — (PDF) [file ppat.1007347.s008.pdf]

S1 Table: Primers used in this study

Primer name	Sequence(5′→3′)	Function
Vip-F	TACCATGGTAATGAACAAGAATAATACTAAATTAAGCAC	Vip cloning
Vip-R	TTCTCGAGCTTAATAGAGACATCGTAAAAATGTAC	Vip cloning
Vip-flag-F	ACTTTAAGAAGGAGATATACCATGGTAATGAACAAGAATAATACT AAATTAAGC	Vip-flag cloning
Vip-flag-R	GTGGTGGTGGTGGTGGTGCTTATCGTCGTCATCCTTGTAATCCTTA ATAGAGACATCGTAAAAATGTAC	Vip-flag cloning
Vip-RFP-Up-F	ACTTTAAGAAGGAGATATACATGAACAAGAATAATACTAAATTAAGC	Vip-RFP cloning
Vip-RFP-Up-R	AGGCCTGCATCTTAATAGAGACATCGTAAAAATG	Vip-RFP cloning
Vip-RFP-Do-F	CTCTATTAAGATGCAGGCCTCTGCAGTCGACGGGCC	Vip-RFP cloning
Vip-RFP-Do-R	GTGGTGGTGGTGGTGGTGCACACTAGTCTTGTACAGCTCGTCCATGC	Vip-RFP cloning
RFP-F	CAAGAGCTCATGCAGGCCTCTGCAGTCGACGGGC	RFP cloning
RFP-R	CGAAAGCTTCTAGATCCAATGACTCTAGATTCG	RFP cloning
Sf-CCP-F	ACACCATGGCATACCCGGCTTCAACATGTCCGAT	Sf-CCP cloning
Sf-CCP-R	ACACTCGAGGCAGCCCGATTTGACACAAACGGGT	Sf-CCP cloning
Dm-CCP-F	GCGCCATGGCACGATGTGAAAGATCTATAGATTTGG	Dm-CCP cloning
Dm-CCP-R	ACACTCGAGGGGCCTCGCACATCCCCTACTG	Dm-CCP cloning
pIZT-SR-C-F	TCGAATTTAAAGCTTGGTACAATGGTAATGTTTATTAAGTGTTTATTATT	Sf-SR-C cloning
pIZT-SR-C-R	AGGCTTACCTTCGAACCGCGGTTCATCATCAACATCGGACTTGA	Sf-SR-C cloning
SRi1-Up-F	TCGAATTTAAAGCTTGGTACATGGGTAGTGTTTTTCTGTTTACCC	SR-C RNAi
SRi1-Up-R	CACATGTATGGAAGGTGGTCCCCCCACTGCTCAAATAGG	SR-C RNAi
SRi1-Do-F	CAGTGGGGGGACCACCTTCCATACATGTGGTAGTAAAAG	SR-C RNAi
SRi1-Do-R	AATGGTGATGGTGATGATGAATGGGTAGTGTTTTTCTGTTTACCC	SR-C RNAi
SRi2-Up-F	TCGAATTTAAAGCTTGGTACTGCTGTCCAGACTTCCTGGACG	SR-C RNAi
SRi2-Up-R	CTAAACCTACACCTATACTGCAGACACTTCAATGGGCAT	SR-C RNAi
SRi2-Do-F	AAGTGTCTGCAGTATAGGTGTAGGTTTAGGAGTAGGCTT	SR-C RNAi
SRi2-Do-R	AATGGTGATGGTGATGATGATGCTGTCCAGACTTCCTGGACGTCT	SR-C RNAi
pAc -SR-C-F	AGACCCCGGATCGGGGTACAATGGTAATGTTTATTAAGTGTTTATTATT	SR-C cloning
pAc -SR-C-R	AGGCTTACCTTCGAACCGCGGTTCATCATCAACATCGGACTTGA	SR-C cloning
pAc-Sf-S2-F	AGACCCCGGATCGGGGTACAATGGCGGACGCAGCTCCAGCCGG	Sf-S2 cloning
pAc-Sf-S2-R	AGGCTTACCTTCGAACCGCGGAGCCTTGAACTCTGAGTAAGGGGAC	Sf-S2 cloning
SR-C-N-F	CTGTTCCAGGGGCCCCTGGGATACTACCCGGCTTCAACATGTCCG	SR-C-N cloning
SR-C-N-R	TCAGTCACGATGCGGCCGCTCCTATCCACTCAGACCTCCAGATTTCTTC	SR-C-N cloning
SR-F-1-F	CTGTTCCAGGGGCCCCTGGGATACCCGGCTTCAACATGTCCG	SR-F-1 cloning
SR-F-1-R	TCAGTCACGATGCGGCCGCTCCTATGACACTTTTGCTGAACTATCACG	SR-F-1 cloning
SR-F-2-F	CTGTTCCAGGGGCCCCTGGGAACGGAATGTGACTTCGAGCTGCCGG	SR-F-2 cloning
SR-F-2-R	TCAGTCACGATGCGGCCGCTCCTAGAGCATGGCTTTGGTGCAGTTCTCC	SR-F-2 cloning
SR-F-3-F	CTGTTCCAGGGGCCCCTGGGAGAAGCCACCACGCCACCAGCTGTT	SR-F-3 cloning
SR-F-3-R	TCAGTCACGATGCGGCCGCTCCTATAATGTAGCTCCACTCAGACCTCC	SR-F-3 cloning
Sf-actin-RT-F	TCCTCCGTCTGGACTTGGC	Actin qRT-PCR
Sf-actin-RT-R	CTTCTCCTTGATGTCACGAACG	Actin qRT-PCR
Sf-SR-RT-F	ATGCTCGAAGCCACCACG	Sf-SR-C qRT-PCR
Sf-SR-RT-R	CGACGCAGACAATGCTACAAC	Sf-SR-C qRT-PCR

pET-SRi-Up-F	AGCGGATAACAATTCCCCTCCTCGTTATACCCTGGTTGG	Se-SR-C RNAi
pET-SRi-Up-R	CAGATCGTAAATAGAGGTTGCAGCGTCCAACAC	Se-SR-C RNAi
pET-SRi-Do-F	CAACCTCTATTTACGATCTGGAAATTATCACCG	Se-SR-C RNAi
pET-SRi-Do-R	AGTGGTGGTGGTGGTGGTGCCTCCTCGTTATACCCTGGTTGG	Se-SR-C RNAi
Hypi-Up-F	AGCGGATAACAATTCCCCTAAGGTGCTCGGAGAAGGAGAG	pET-Hypi
Hypi-Up-R	CACAGCAACGAGGAGCACGCCGAACGACCAG	pET-Hypi
Hypi-Do-F	GCGTGCTCCTCGTTGCTGTGGACATCCTTAGCC	pET-Hypi
Hypi-Do-R	AGTGGTGGTGGTGGTGGTGCAAGGTGCTCGGAGAAGGAGAG	pET-Hypi
Se-actin-RT-F	ATCCTCCGTCTGGACTTGGC	Actin qRT-PCR
Se-actin-RT-R	CTTCTCCTTGATGTCACGCACG	Actin qRT-PCR
Se-SR-RT-F	AACCTATAACAGTGTCCGTTGAAG	Se-SR-C qRT-PCR
Se-SR-RT-R	AATGTTGCTCCACTCAGTCCTC	Se-SR-C qRT-PCR
pJF-Sf-SR-C-F	ATCCTTTACTTCAGGCGGCCGCGGCTCGAGCAAAATGTTTATTAA GTGTTTATTATTTTTATTTG	Sf-SR-C cloning
pJF-Sf-SR-C-R	TCCTTCACAAAGATCCTCTAGATTATTCATCATCAACATCGGAC	Sf-SR-C cloning
