# Supplementary material for: Scavenger receptor-C acts as a receptor for Bacillus thuringiensis vegetative insecticidal protein Vip3Aa and mediates the internalization of Vip3Aa via endocytosis
Source: PLoS Pathog. 2018 Oct 4;14(10):e1007347. doi: 10.1371/journal.ppat.1007347 (PMC6191154; doi:10.1371/journal.ppat.1007347)
Supplement: S2 Table — (PDF) [file ppat.1007347.s009.pdf]

S2 Table: Plasmids used in this study

Plasmids	Relevant characteristics	Source
pET-28a(+)	Expression vector, Kan^r, C/N-terminal His tag	Novagen
pET-Vip	Vip3Aa gene cloned into pET-28a(+), His tag binding C-terminal of Vip3Aa	This study
pET-Vip-flag	Vip3Aa gene cloned into pET-28a(+), Flag-His tag binding C-terminal of Vip3Aa	This study
pET-Vip-RFP	RFP gene cloned into pET-Vip, RFP binding C-terminal of Vip3Aa	This study
pET-RFP	RFP gene cloned into pET-28a(+), His tag binding C-terminal of RFP	This study
pET-ChiB-flag	ChiB gene cloned into pET-28a(+), Flag-His tag binding C-terminal of ChiB	Lab collection
pET-Sf-CCP	Sf-CCP cloned into pET-28a(+), His tag binding C-terminal of Sf-CCP	This study
pET-Dm-CCP	Dm-CCP cloned into pET-28a(+), His tag binding C-terminal of Dm-CCP	This study
pIZT/V5-His	Expression vector, $Zeocin^r$, C-terminal V5-His tag	Invitrogen
pIZT-SR-C	Sf-SR-C gene cloned into pIZT/V5-His, V5-His tag binding C-terminal of Sf-SR-C	This study
pIZT-SRi1	Fragment of Sf-SR-C gene(294-803) and the reverse complemented Fragment of SR-C (693-294) cloned into pIZT/V5-His	This study
pIZT-SRi2	Fragment of Sf-SR-C gene(1081-1590) and the reverse complemented Fragment of SR-C (1480-1081) cloned into pIZT/V5-His	This study
pAc5.1/V5-His B	Expression vector, C-terminal V5-His tag	Invitrogen
pAc-Sf-SR-C	Sf-SR-C gene cloned into pAc5.1/V5-His B, V5-His tag binding C-terminal of Sf-SR-C	This study
pAc-Sf-S2	Sf-S2 gene cloned into pAc5.1/V5-His B, V5-His tag binding C-terminal of S2	This study
pGEX-6P-1	Expression vector, Amp^r, N-terminal GST tag	Lab collection
pGEX-SR-C-N	Extracellular sequence of Sf-SR-C gene (SR-C-N) cloned into pGEX-6P-1, GST tag binding N-terminal of SR-C-N	This study
pGEX-SR-F-1	SR-F-1 gene fragment cloned into pGEX-6P-1, GST tag binding N-terminal of SR-C-F-1	This study
pGEX-SR-F-2	SR-F-2 gene fragment cloned into pGEX-6P-1, GST tag binding N-terminal of SR-C-F-2	This study
pGEX-SR-F-3	SR-F-3 gene fragment cloned into pGEX-6P-1, GST tag binding N-terminal of SR-C-F-3	This study
pJFRC2-10XUAS-IVS-mCD8::GFP	Refinement of Tools for Targeted Gene Expression in Drosophila.	Addgene
pET-Hypi		This stydy
pET-Se-SRi	Fragment of Se-SR-C gene(1-870) and the reverse complemented Fragment of SR-C (718-1) cloned into pET28a(+)	This study

Amp^r: ampicillin resistance, Kan^r: kanamycin resistance, $Zeocin^r$: zeocin resistance
